# A simplified material flow analysis employing local expert judgment and its impact on uncertainty

**DOI:** 10.1007/s10163-023-01660-5

**Published:** 2023-05-02

**Authors:** Wutyi Naing, Hidenori Harada, Shigeo Fujii, Chaw Su Su Hmwe

**Affiliations:** 1 Graduate School of Asian and African Area Studies, Kyoto University, 46 Yoshida-Shimoadachi-Cho, Sakyo, Kyoto 606-8501 Japan; 2 Graduate School of Global Environmental Studies, Kyoto University, Kyoto, Japan; 3 Department of Chemical Engineering, Mandalay Technological University, Patheingyi, Myanmar

**Keywords:** Material flow analysis, Local expert judgment, Waste management, Nitrogen, Phosphorus, Low- and middle-income countries

## Abstract

Material flow analysis (MFA) is an effective tool for waste management, but low- and middle-income countries lack essential data for MFA. This study proposed a simplified MFA (sMFA) utilizing local expert judgment (LEJ) and examining the impact of simplification on its uncertainty. A stochastic sMFA model was developed for nitrogen and phosphorus in urban Mandalay, Myanmar. This model was compared with the intensive MFA (iMFA) model employing intensive surveys for primary data collection. For the total loadings to the environment, the medians of the sMFA were higher by 3% and 11%, respectively, for nitrogen and phosphorus than those of the iMFA. The widths of the 80% confidence intervals of these loadings in the sMFA, normalized by those in the iMFA, were − 0.05 and − 0.11, respectively. The three largest flows to the environment were the same for the two models: on-site sanitation effluent/leakage, greywater, and industrial wastewater. Large median gaps between the models were observed for industrial wastewater, fecal sludge, and human excreta, associated with informal waste management, whereby LEJ did not work well. Overall, the sMFA demonstrated a good estimation of nitrogen and phosphorus flows with limited increase of uncertainty, still requiring focused attention on informal waste streams.

**Graphical abstract:**

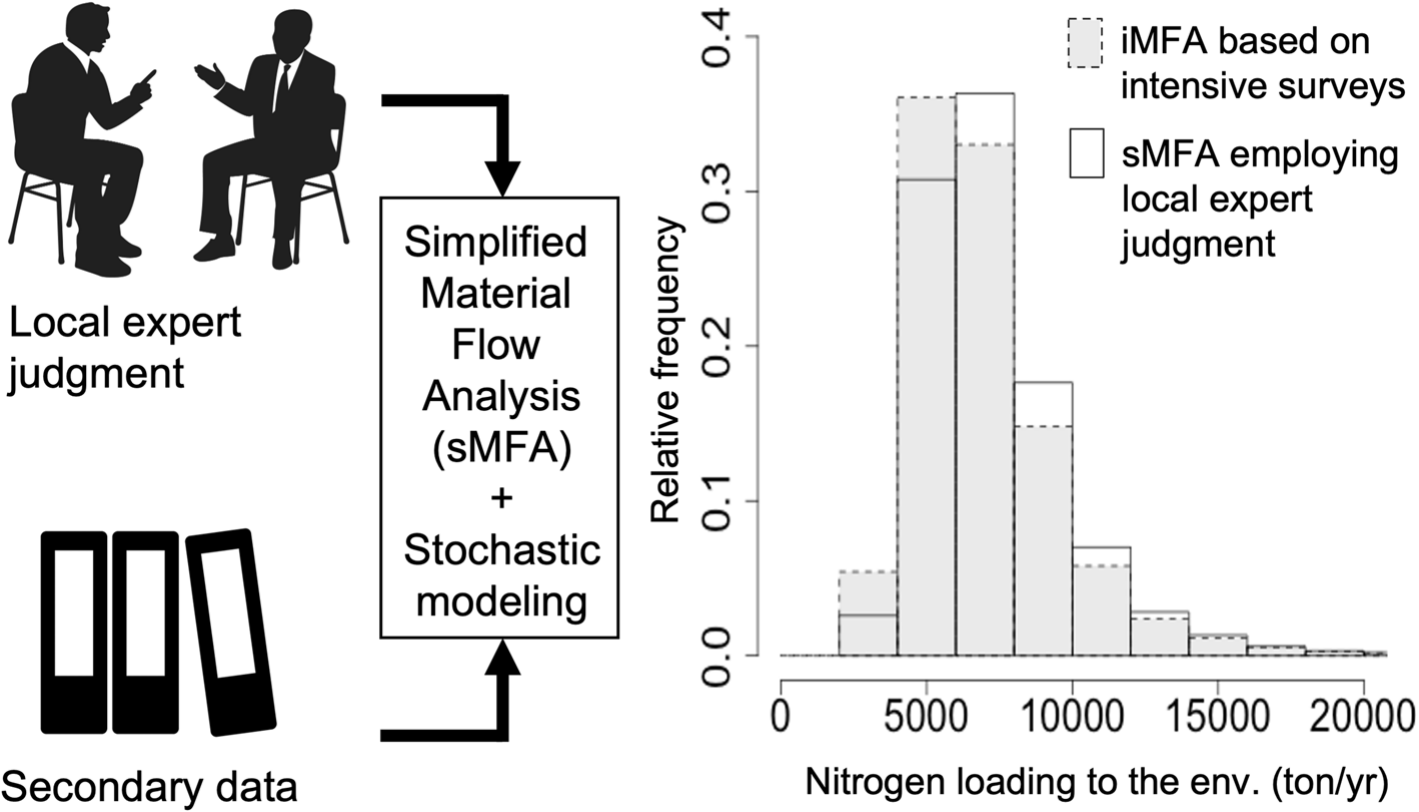

**Supplementary Information:**

The online version contains supplementary material available at 10.1007/s10163-023-01660-5.

## Introduction

Along with rapid urbanization and population growth, anthropogenic organic waste and wastewater have become a great threat to the environment owing to unplanned urban waste and wastewater management, especially in low- and middle-income countries. It is essential to quantify anthropogenic organic waste and wastewater flow to design an effective waste and wastewater management plan. Although physical modeling is a powerful tool to analyze the detailed dynamics of target materials in the system, material flow analysis (MFA) is an alternative and attractive tool to simply describe the flow of the target materials for resource, waste, and environmental management in a region [1]. MFA balances input and output, enabling them to establish flows of waste and wastewater between components, estimate environmental loadings, and identify their sources within a system defined in space and time through a systematic assessment. Based on MFA, waste and wastewater management strategies can be evaluated using pollutant flows, resource recovery potentials, and environmental loading.

MFA has been used for waste management since the late twentieth century, mainly in high-income countries, such as Switzerland [2], the United States [3], Sweden [4], and Danube countries [5]. A reliable MFA is developed through abundant information, such as statistics on demographics, water and goods consumption, unit generation for target materials, and waste and wastewater management practices. Although such large and essential datasets for MFA development may exist in high-income countries, they often lack or are inaccurate in low- and middle-income countries. Hence, MFA development is a time-and money-consuming task that requires additional resources for data collection in areas where required information has not been previously assessed [1].

MFA has been also applied in some of low- and middle-income countries by putting great effort into data collection. For example, an MFA in Tunja, Colombia, was developed based on a 575-household survey [6]; an MFA in Bangkok, Thailand, based on a 300-household survey [7]; and another MFA in Kuala Lumpur, Malaysia, based on a 450-household survey [8]. These MFA developments often accompany with chemical composition surveys on waste and wastewater. Thus, besides using data from other regions, the city-wide scale application of an MFA requires a resource-intensive primary data collection campaign, which burdens the city to identify waste and wastewater flows in low- and middle-income countries.

Knowledge of local expert can be used to develop an MFA as an alternative to the primary data collected by such resource-intensive data collection campaigns. Here, we define local expert judgment (LEJ) as the subjective assessment of a process where enough data are not available to assess it statistically. In 2007, LEJ was used in a systematic manner to assess the nutrient flows in septic tanks in Vietnam [9]. Experts were asked to provide a range of estimated values of nutrient transfer coefficients in septic tanks. Furthermore, the LEJ was used to assess the plausibility of the MFA parameters and calculated flows [10, 11]. LEJ is not only effective for the plausibility assessment of MFAs, but it also facilitates an efficient MFA development using LEJ as an alternative to essential primary data to be collected at a city scale. However, the previous studies [12, 13] showed the importance of accounting for data uncertainty in MFAs. This can be especially important in MFAs employing LEJ as an alternative to reliable primary data, which potentially increases the uncertainty of the MFA. Supposed that the uncertainty of MFA employing LFJ is limited to a reasonable degree, the systematic use of LEJ could simplify the development process of MFA, which has been conventionally based on labor-intensive data collection, contributing to the effective understanding of waste and wastewater flows for urban waste and wastewater management, especially in low- and middle-income settings. However, little is known about the impact of LEJ on the uncertainty of MFAs.

We propose a simplified MFA (sMFA) by employing LEJ as an alternative to primary data to be collected by a resource-intensive data collection campaign. The present paper aimed to examine the impact of the simplification on the uncertainty of the sMFA and to validate the effectiveness of the sMFA. A stochastic sMFA model was developed for nitrogen and phosphorus in urban Mandalay, Myanmar, a city in which essential data were lacking for an MFA. We also developed an intensive MFA (iMFA) model based on intensive surveys for primary data collection. The two MFA models were compared to examine the impact of the simplification on the estimation of waste and wastewater flows and total environmental loading of phosphorus and nitrogen, so that we could evaluate the uncertainty of sMFA employing LEJ against iMFA, and explore sMFA’s potential and limitation to complement iMFA.

## Materials and methods

### Study area information

Mandalay, the capital of Mandalay region, is the second largest and most densely populated city in Myanmar. It is on the east bank of the Irrawaddy River on approximately 80 m above sea level and in the central dry zone of the country with hot semi-arid climate and around 800 mm of annual precipitation. The city occupies 912.7 km^2^ with a population of 1.7 million [14]. This study selected five urban townships: Aung Myae Tharzan, Chan Aye Tharzan, Chan Mya Tharzi, Mahar Aung Myae, and Pyigyi Dagon, which cover 95% of the city’s urban areas (108 km^2^) and 1.2 million people with 0.8 km^2^ of agricultural land [14].

Mandalay fully relies on on-site sanitation, which are mainly septic tanks receiving blackwater only. As of 2017, among emptying-experienced on-site sanitation in urban Mandalay, 32.8% were formally emptied and fecal sludge was transported to the designated disposal pond, while the remaining were emptied as informal business and majority of them were illegally dumped [15]. Fecal sludge is one of major issues in the city. No domestic wastewater treatment plant exists, and a combined open drainage system is used for greywater management in the city. Although there are three industrial zones inside the study area, there are no centralized industrial wastewater treatment plant apart from some stabilization ponds running by a few industries in the area. There are two landfill sites for solid waste management. As of 2016, the solid waste collection of the city development committee covered 80% of the city [16].

### Procedure of the iMFA and sMFA model development

#### Defining system boundary

**Fig. 1:**
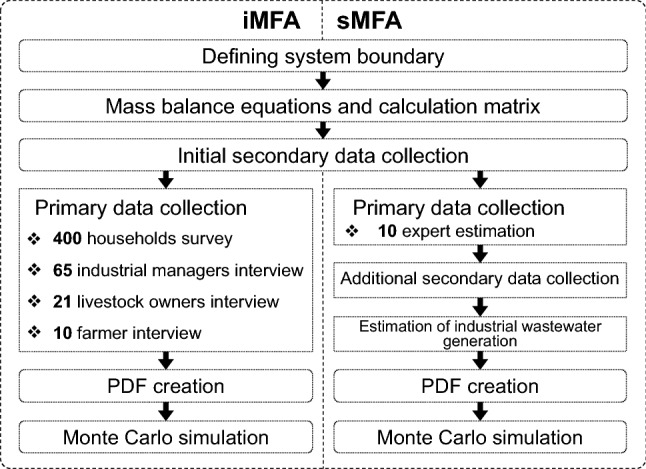
Developmental procedures of the intensive (iMFA) and simplified material flow analysis (sMFA) models in a stochastic manner

Figure 1 shows the procedures of the iMFA and sMFA model development for nitrogen and phosphorus flow. The system boundary was the same for the two models, as shown in Figure S1. The geographical boundaries were five townships in urban Mandalay. The models included five components (X_*j*_) inside the system boundary: household (X_1_), on-site sanitation (X_2_), livestock (X_3_), agriculture (X_4_), and industry (X_5_). The other seven components were outside the boundary: market (X_6_), fecal sludge disposal pond (X_7_), solid waste disposal sites (X_8_), soil and water environment (ground soil, drainage, and surface water in this study; X_9_), atmosphere (X_10_), outside livestock (X_11_), and outside agriculture (X_12_).

#### Mass balance equations and waste flow calculation matrix

The model equations for nitrogen and phosphorus flows were the same for the two models, except for fecal sludge from on-site sanitation, following the mass conservation law and modifying the MFA models of previous studies [17, 18]. Briefly, the model consisted of 24 reaction flow processes (P_*i*_). The net reaction rate of a component within the boundary was zero [Eq. (1)]. The sum of the reaction transfer factors for each reaction flow process is considered as the mass balance [Eq. (2)]. The details of each reaction flow process, reaction transfer factor, and reaction rate are summarized in Table S1 with working examples of Eqs. (1) and (2)

1 $${r}_{j}=\sum_{i}\left( {\beta }_{i,j}\cdot {\rho }_{i}\right)=0$$

2 $${f}_{i}=\sum_{j}\left( {\beta }_{i,j}\right)=0,$$

where *r*_*j*_ is the net reaction rate for the component *j* within the system boundary (ton/year), *β*_*i,j*_ is the transfer factor to the component *j* through reaction flow process *i* (−), ρ_*i*_ is the reaction rate of reaction flow process *i* (ton/year), and *f*_*i*_ is the sum of reaction transfer factors for the process* i* (−).

#### Initial secondary data collection

The initial secondary data collection was the same for both models. The secondary data to estimate the reaction rate of reaction flow processes (ρ_*i*_) were collected from the city statistics (e.g., land areas, human population, on-site sanitation unit, livestock population and production, and industrial production), as well as local survey reports and other literature (nitrogen and phosphorus loading and concentration of waste and wastewater), as summarized in Table S2.

#### Primary data collection

For the data which we could not find appropriate secondary data, primary data were collected for the iMFA and sMFA differently, which are mainly data for transfer factors (*β*_*i*,*j*_) and reaction processes (ρ_*i*_), as shown in Table S3. The primary data collection was conducted in two different ways for iMFA and sMFA during September 2017–March 2018.

The iMFA employed an intensive survey to collect the data of the actual practices of waste and wastewater management through interviewing to nearly 500 respondents: water consumption, organic waste management, and wastewater management practices of householders (*n* = 400), livestock owners (*n* = 21), farmers (*n* = 10), and industrial factory managers (*n* = 65). The iMFA did not use any of LEJ explained below. The detailing of the surveyed items is shown as ‘intensive survey’ in Table S3.

In contrast, the sMFA collected local expert knowledge by interviewing the same contents to ten experts who had more than 5 years of working experience in each sector: experts from the waste management sector (*n* = 6), city development sector (*n* = 3), and environmental sector (*n* = 1). Without sharing any intensive survey data mentioned above, experts were requested to subjectively conjecture the values of each parameter based on their experience (i.e., LEJ). Of the ten experts, at least nine could give their LEJ for each parameter, except industrial wastewater generation and livestock water consumption.

#### Additional secondary data collection and parameter estimation for the sMFA

Since LEJ was not obtained for livestock water consumption, it was estimated based on other studies [19, 20]. Industrial wastewater generation was estimated using the guideline values of WHO [21] and OECC [22] based on the available secondary data. The details are presented in Tables S3 and S4.

#### Defining PDFs for stochastic analysis using Monte Carlo simulation

Probability density functions (PDFs) were created for the parameters of the iMFA and sMFA based on the data collected for each (Tables S2, S3, and S4). Nitrogen and phosphorus concentrations and their loading data and proportional data were fitted into lognormal and beta distributions, respectively. Other continuous data with *n* ≥ 5 were fitted into a normal distribution, whereas those with *n* < 5 were fitted into a triangular distribution. In total, parameter fitting of 47 PDFs was conducted using the fitdisplus package in R version 4.0.0 (R Core Team) [23]. After defining the PDFs for each parameter, the process flows were calculated in a stochastic manner separately for the iMFA and sMFA by the Monte Carlo simulation with 100,000 trials to examine the variability of the estimates.

#### Uncertainty analysis

The uncertainty of the sMFA compared with the iMFA was examined by comparing the nitrogen and phosphorus loadings of each flow to the soil and water environment between the two stochastic models. The difference between the medians of the iMFA and sMFA was normalized by the median of the iMFA, as shown in Eq. (3)

3 $$n\Delta {\mathrm{med}}_{r(i,9)}= \frac{\left|{\mathrm{median}}_{\mathrm{sim},r(\mathrm{i},9)}-{\mathrm{median}}_{\mathrm{int},r(\mathrm{i},9)}\right|}{{\mathrm{meidan}}_{\mathrm{int},r(\mathrm{i},9)}},$$

where *n∆*med_*r*(*i*,9)_ is the normalized median difference of pollution loading from reaction flow process *i* to the soil and water environment (X_9_) between the sMFA and iMFA; median_sim*,r(i*,9)_ and median_int*,r(i*,9)_ are the medians of pollution loading from reaction flow process *i* to the soil and water environment (X_9_) for the iMFA and sMFA, respectively.

In addition, to compare the variability of the iMFA and sMFA, the width of the 80% confidence interval (CI) of the loadings in sMFA was estimated by the percentile values of Monte Carlo simulation, and it was normalized by dividing the width of 80%CI for simplified MFA by that for intensive MFA, as shown in Eq. (4)

4 $${n80\%\mathrm{CI}}_{r(i,9)}= \frac{{w80\%\mathrm{CI} }_{\mathrm{sim},r(i,9)}}{{w80\%\mathrm{CI} }_{\mathrm{int},r(i,9)}},$$

where *n*80*%*CI_*r*(*i*,9)_ is the normalized width of 80% CI of pollution loading from reaction flow process *i* to the soil and water environment (X_9_) for the sMFA; *w*80%CI_sim*,r*(*i*,9)_ and *w*80%CI_int,*r*(*I*,9)_ a the width of 80% CI of pollution loading from reaction flow process *i* to the soil and water environment (X_9_) for iMFA and sMFA, respectively.

### Sensitivity analysis

The sensitivity of each parameter to the total loading to the soil and water environment was analyzed following the methodology of a previous study [24]. Briefly, all the parameter values were fixed at the 50^th^ percentile (i.e., p50) of each PDF parameter, and the loading was estimated as p50. Keeping all the parameters at p50 except one parameter, the value of one parameter was changed to the 10^th^ percentile (p10) and the 90^th^ percentile (p90) of the PDF. The calculated total loadings were obtained as p10 and p90, respectively. The sensitivity of the parameter was expressed as the ratio of the total loading change as p10:p50 and p90:p50.

## Results and discussion

### Waste management practice in Mandalay

Table S5 summarizes the waste management practices in the study area, based on the intensive survey results. Briefly, 98% of excreta is discharged to on-site sanitation, with the remainder being directly discharged into the environment. This proportion is slightly higher than the proportion in urban areas of Mandalay region (92.3%) [25]. Of the greywater, 99% is discharged to open drainage. Of the kitchen waste, 80% is transported to disposal sites; the percentage is the same magnitude to the proportion in urban areas of Mandalay region (70.6%) [25]. Of the emptied fecal sludge from on-site sanitation, only 20% is formally disposed of at the designated pond, and the remaining is discharged to the environment. Similarly to the management of many cities in low- and middle-income countries [26], fecal sludge management is poor in the area. Of livestock manure, 57% is used in agricultural activities outside the study area, whereas the remaining is discharged to the environment. Of the agricultural residues, 84% of rice residues and 100% of vegetable residues are left in agricultural fields, whereas 16% of rice residues are used for livestock feeding. Of industrial organic solid waste, 72% and 11% are used for livestock feeding outside and inside the study areas, respectively; 11% is disposed at disposal sites; the remaining is discharged to the environment. All industrial wastewater is directly discharged into the environment.

### Comparison of local expert judgment and intensive survey on waste management practice

**Fig. 2:**
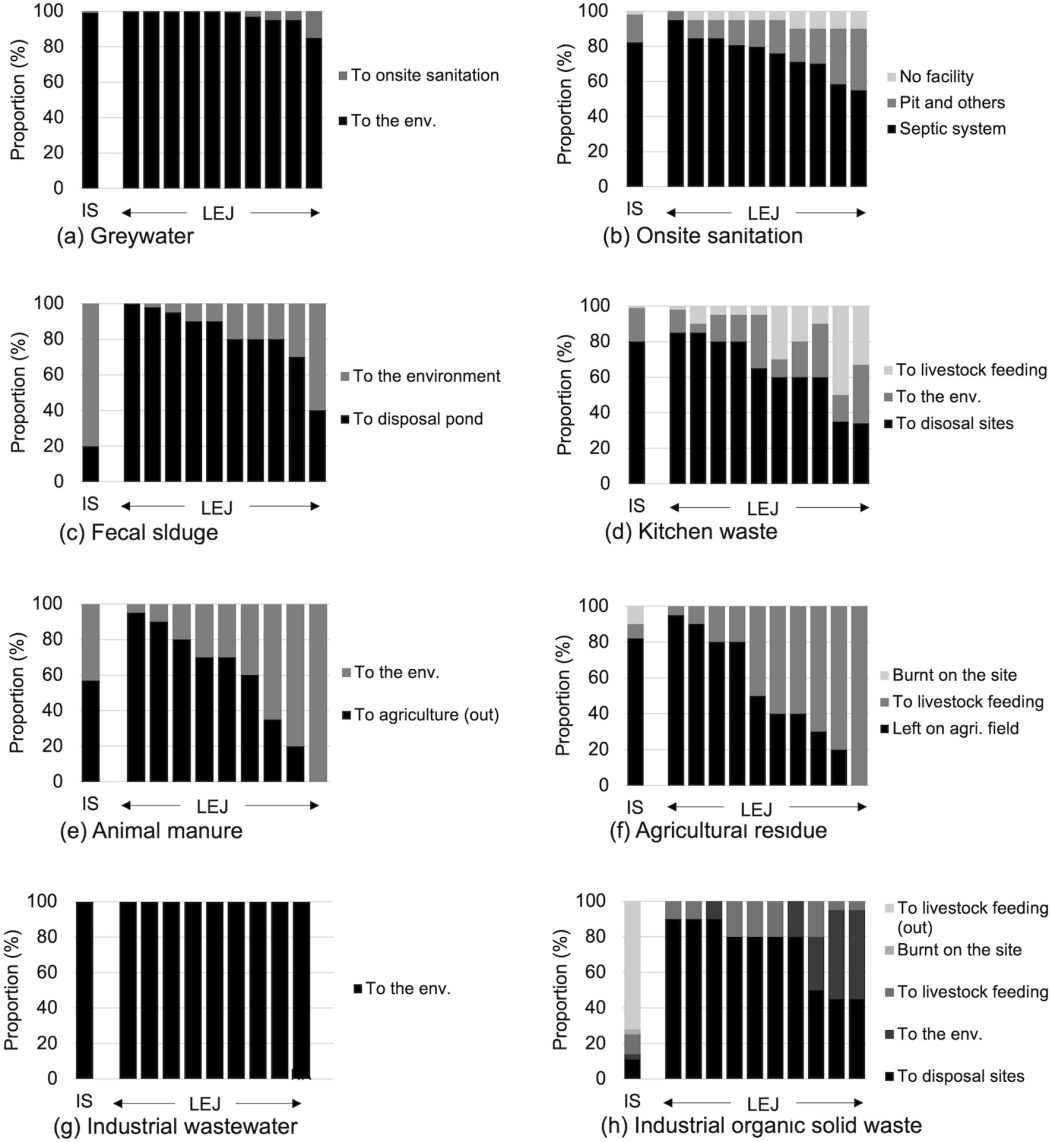
Comparison of primary data on waste management practices between an intensive survey (IS) and local expert judgment (LEJ). “The environment” means the soil and water environment. “(Out)” means the activities outside of the study area. The results from LEJ were arranged in decreasing order of the major items for each of the panels [**a**–**h**]

The LEJ on waste management practices was compared with the intensive survey (IS), as shown in Fig. 2 and Table S5. Although the LEJs varied among ten local experts, the averages of LEJs and IS were of the same magnitude for on-site sanitation (81% and 82% for the septic system), greywater (97% and 99% to the environment), livestock manure (58% and 57% for agriculture), kitchen waste (65% and 80% to disposal sites), and industrial wastewater (100% and 100% to the environment, respectively).

In contrast, the averages of LEJs and IS were substantially different for agricultural residues (53% and 92% left on agricultural fields), fecal sludge (82% and 20% to disposal ponds), and industrial organic solid waste (73% and 11% to disposal sites, respectively). For agricultural residues, the selection of experts might create a gap between the LEJ and IS. According to a previous study [27], 8–15 local experts are necessary, and the selection of experts is still important. In the present study, although ten experts were used, seven of them were selected from the waste management sector, and they may not have had enough knowledge of local farmer practices regarding agricultural residues. For fecal sludge, the experts were selected from the sector responsible for fecal sludge emptying. However, the city has widespread illegal behavior concerning fecal sludge emptying; 65% of fecal sludge was emptied illegally in the city [15]. This intensive informal activity possibly resulted in a discrepancy between the LEJ and IS. Another possible reason for this discrepancy is response bias with social desirability [28], in which the responsible experts potentially desired formal emptying activities in the city.

Of the industrial organic solid waste, the large gap could be explained by two possible reasons. First, IS estimation was based on interviews with industrial managers. Potentially due to the response bias with dissimulation [28], they might have reported less disposal waste to reduce disposal fees, which was determined based on the disposal amount. Second, the local experts, responsible for waste management in the city, possibly missed the resource recovery of private sectors beyond the city area. No expert answered industrial solid waste burning on the site and the resource recovery actions outside of the boundary, although 3% and 72% of industrial solid waste were burned on-site and used for livestock outside the study area according to IS. In contrast, experts had a good estimate for industrial solid waste resource recovery inside the city area: 11% and 11% by LEJ and IS, respectively. These results partly imply the good applicability of LEJ to estimate waste and wastewater management practices, although a special attention is required for following: the proper selection of experts' expertise, response bias, illegal business, disposal fees, and recycling outside of the experts’ jurisdiction area.

### Baseline estimation of N and P flows based on the iMFA

**Fig. 3:**
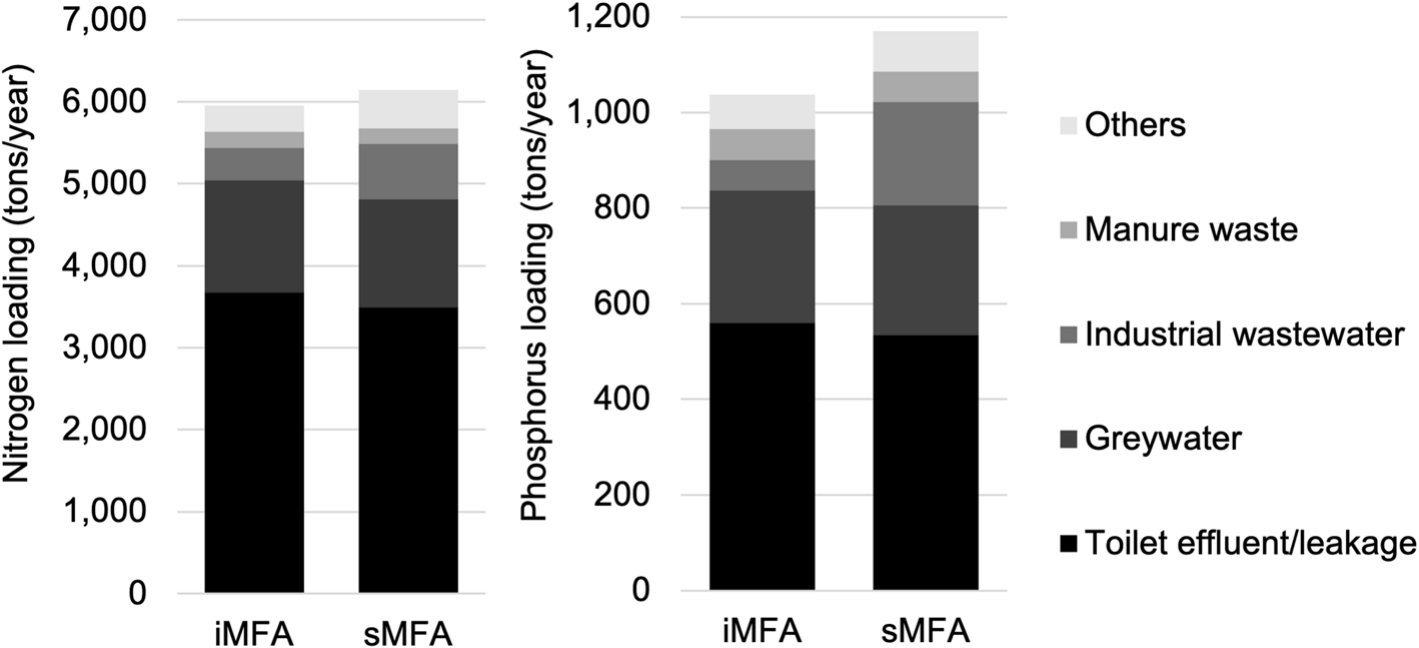
Nutrient loading to the soil and water environment from the stochastic modeling of the intensive (iMFA) and simplified material flow analysis (sMFA) as medians with 100,000 iterations (left: nitrogen; right: phosphorus)

The baseline nitrogen and phosphorus flows were estimated based on the medians of stochastic iMFA, and the details are shown in Figure S2(a) and (b). As summarized in Fig. 3, the total pollution loading to the soil and water environment (i.e., the environment) was estimated at 5,981 tons of nitrogen per year (tons/year) and 1,040 tons of phosphorus per year (tons/year), equivalent to 55 tons of nitrogen per year per square kilometer (tons/year/km^2^) and 9.6 tons of phosphorus per year per square kilometer (tons/year/km^2^). These loadings are of the same magnitude as those of other cities in Southeast Asia, such as Bangkok [7], Hue [29], and urban Hanoi [30]. Similarly to the above three cities of the total loading to the environment, the majority (85% of nitrogen and 81% of phosphorus) were derived from toilet effluent/leakage from on-site sanitation for nitrogen (3,670 tons/year) and phosphorus (560 tons/year) and greywater from households for nitrogen (1,369 tons/year) and phosphorus (277 tons/year). In addition, industrial wastewater from industries accounted for 7% (395 tons/year) of the total nitrogen loading and 6% (63 tons/year) of the total phosphorus loading to the environment. Agricultural runoff was limited, as the study sites were urban areas, and agricultural activity was not active. Of the total nitrogen and phosphorus input to the boundary, 40% was discharged into the environment. Thus, improvement of toilet/on-site sanitation management would be the most crucial challenge to establish sound nutrient cycle in urban Mandalay.

**Fig. 4:**
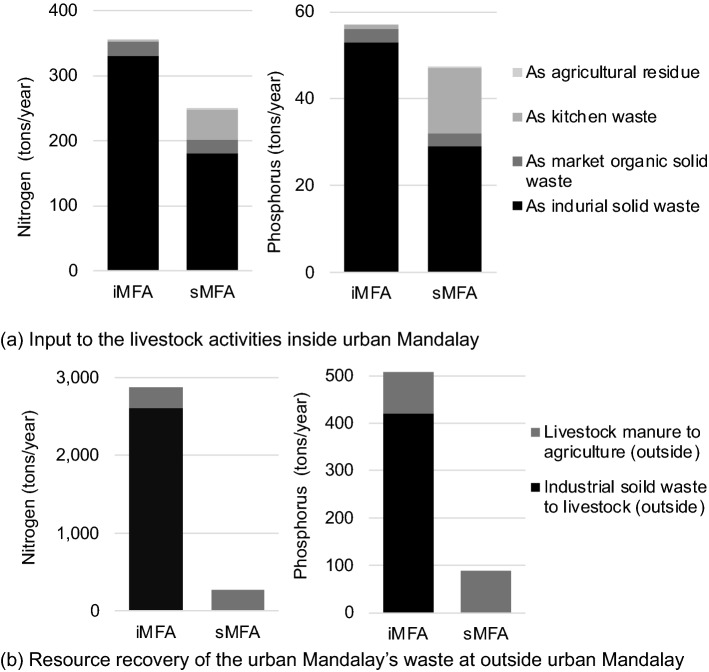
Resource recovery activities inside and outside Mandalay from the stochastic modeling of the intensive (iMFA) and simplified material flow analysis (sMFA) as medians with 100,000 iterations (left: nitrogen; right: phosphorus)

Focusing on resource recovery inside the study area, livestock inside the study area played a major role in recycling waste flows (Figure S2), similarly to sub-urban Hanoi [30]. As shown in Fig. 4a, livestock received 330 tons of nitrogen per year (tons/year) and 53 tons of phosphorus per year (tons/year) of organic solid wastes from industry for livestock feeding based on iMFA, accounting for 36% nitrogen and 27% phosphorus of the total input to livestock inside urban Mandalay and 519 tons of nitrogen per year (tons/year) and 128 tons of phosphorus per year (tons/year) of commercial feed from the market. Conversely, the waste resources generated inside the study area were actively used outside the boundary. Livestock outside the study area received 2,610 tons of nitrogen per year (tons/year) and 420 tons of phosphorus per year (tons/year) of organic solid wastes from the industry for feeding (Fig. 4b), corresponding to 19% nitrogen and 19% phosphorus of the total output from urban Mandalay. Along with livestock, agriculture outside the study area also contributed to the resource recovery of the wastes generated inside urban Mandalay: 270 tons of nitrogen per year (tons/year) and 88 tons of phosphorus per year (tons/year) of recovered manure from livestock inside the area (Fig. 4b), corresponding to 2% nitrogen and 4% phosphorus of the total output from urban Mandalay. Thus, the resource recovery of the waste generated inside urban Mandalay was mainly achieved by livestock and agricultural activities outside urban Mandalay rather than that inside.

### Comparison of major pollution sources and resource recovery between the sMFA and iMFA

As shown in Fig. 3, the medians of the total nitrogen and phosphorus pollution loading to the environment from the sMFA were 6,144 tons of nitrogen per year (tons/year) and 1,171 tons of phosphorus per year (tons/year), respectively; 3% and 11% higher than those of the iMFA. Corresponding to the baseline estimation by the iMFA, the four largest flows to the environment by the sMFA were effluent/leakage from on-site sanitation for nitrogen (4,390 tons/year) and phosphorus (534 tons/year), greywater from household for nitrogen (1,323 tons/year) and phosphorus (271 tons/year), wastewater from industry for nitrogen (671 tons/year) and phosphorus (217 tons/year), and manure from livestock inside the area for nitrogen (195 tons/year) and phosphorus (64 tons/year). Overall, the sMFA demonstrated the effectiveness of estimating pollution loading to the environment.

For these four major flows to the environment, the median estimates of the sMFA were similar to those of the iMFA within a 5% difference, except for industrial wastewater. The nitrogen and phosphorus loadings to the environment as industrial wastewater were 70% and 244% higher than those of the iMFA, respectively. This overestimation can be explained as follows: (1) as mentioned above, the estimation of industrial wastewater generation in the sMFA relied on the guideline values [21, 22] created outside Myanmar as the expert could not answer any LEJ during our interview; this might result in a significant error in the industrial wastewater flows estimated in the sMFA. (2) The gap in industrial wastewater flows between the iMFA and sMFA could be related to underestimating the iMFA caused by the response bias with dissimulation [28], similar to the industrial organic waste disposal in the previous section. Potentially, industrial managers provided information on lower water consumption and wastewater discharge than the actual values to avoid production capacity tax and waste disposal charges.

As shown in Fig. 4a, for resource recovery, industrial solid waste recycling by livestock inside the area by the sMFA was 40% lower than that of the iMFA. Kitchen waste recycling as animal feeding in livestock by the sMFA (46 tons/year for nitrogen and 15 tons/year for phosphorus) was higher than that by the iMFA (3 tons/year for nitrogen and 1 ton/year for phosphorus). The remaining resource recovery options within the system boundary were similar to those of the iMFA. As seen in Fig. 4b, no waste was used on livestock outside the area by the sMFA, despite being one of the major recycling flows by the iMFA. As mentioned in the previous section, experts could not recognize the resource recovery action of industrial waste by the livestock outside. In contrast, the resource recovery of livestock manure to agriculture outside the area showed similar results for the iMFA and sMFA. As many low- and middle-income countries still face challenges in establishing good industrial statistics [31], the lack of good local industrial statistics could be a challenge to increase the accuracy of the estimation by both the iMFA and sMFA.

### Comparison of pollution loading variability between the iMFA and sMFA

**Fig. 5:**
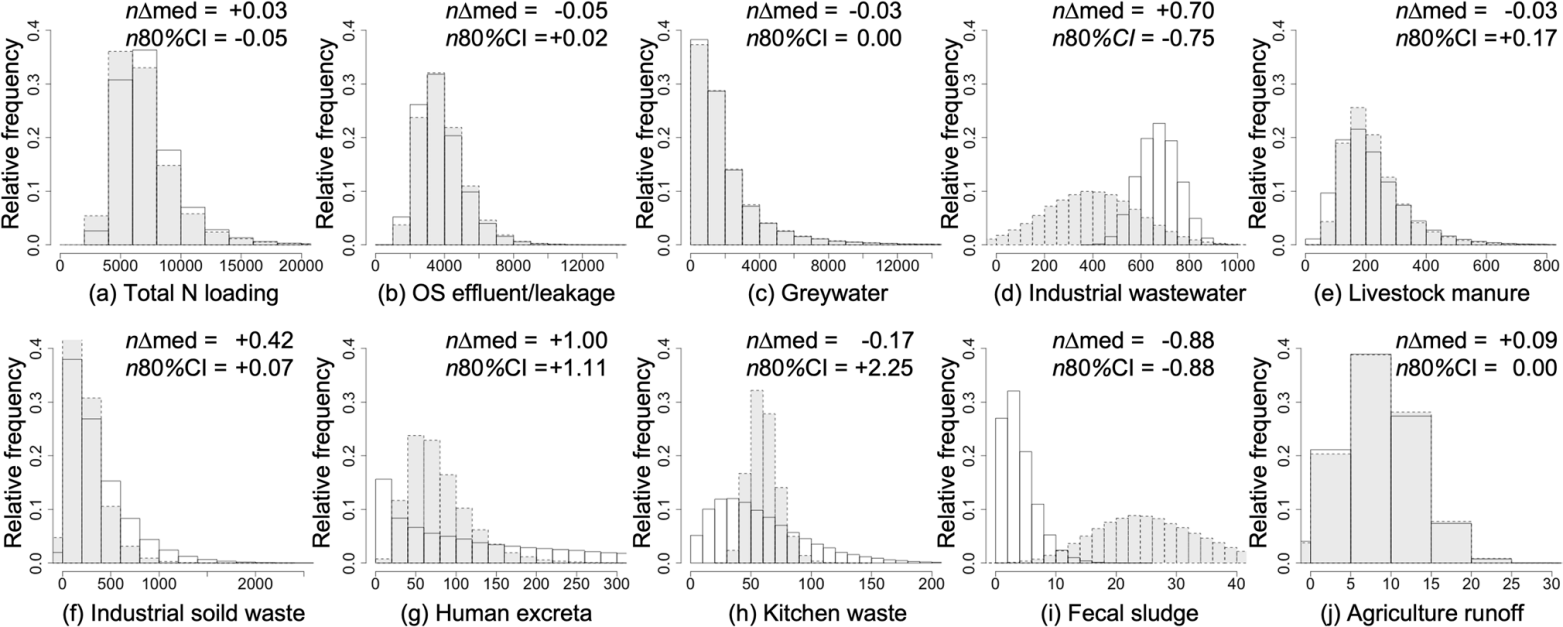
Histograms of nitrogen loadings to the soil and water environment of stochastic intensive (iMFA) and simplified material flow analysis (sMFA) with 100,000 iterations (ton/year). Gray histograms were referred to iMFA and white ones to sMFA. OS means on-site sanitation. *n*∆med means normalized median difference and *n*80%CI means normalized width of 80% confidence interval

The variability of total and individual nitrogen loadings to the environment by the iMFA and sMFA was examined by the normalized median difference and the normalized 80% CI of the estimated loadings of the sMFA compared with the iMFA (Eqs. 3 and 4). Overall, the total loadings of the iMFA and sMFA were 5,214; 6,419; and 8,059 tons of nitrogen per year (tons/year) and 5,595; 6,805; and 8,446 tons of nitrogen per year (tons/year), and 913; 1,117; and 1,380 tons of phosphorus (tons/year) and 1,078; 1,283; and 1,549 tons of phosphorus (tons/year) (1st; 2nd; and 3rd quartiles, respectively). The normalized median differences of total loading were, + 0.03 and 0.11 for nitrogen and phosphorus, respectively (Figs. 5 and S4). The normalized width of 80% CI of the total loading was − 0.05 and − 0.11 for nitrogen and phosphorus, respectively. These indicate the limited impact of the simplification of MFA and the validity of the sMFA to estimate the total loading to the environment.

The distribution of the individual nitrogen loadings from each component to the environment are shown as histograms in Fig. 5 (refer to Figure S4 for phosphorus). Overall, the distribution patterns of individual nutrient flows were similar between the iMFA and sMFA, except for fecal sludge and industrial wastewater. The normalized median differences of on-site sanitation effluent/leakage, greywater, and livestock manure for nitrogen, which were three of the four major flows, were − 0.05, − 0.03, and − 0.03, respectively; those of the same three flows for phosphorus were − 0.05, − 0.02, and − 0.03, respectively. The normalized widths of 80% CI of these three major flows for nitrogen were + 0.02, 0.00, and + 0.17, respectively, and those for phosphorus were + 0.01, 0.00, and + 0.17, respectively. Such results indicate the limited impact of MFA simplification and the good performance of the sMFA in terms of the loading estimation and the estimates variability of these three major flows.

In contrast, a large gap was observed between the iMFA and sMFA for industrial wastewater, which is the remaining of the four major flows. The normalized median differences of the loading of industrial wastewater were + 0.70 and + 2.44 for nitrogen and phosphorus, respectively; the normalized widths of 80% CI of the loading were − 0.75 and − 0.70 for nitrogen and phosphorus, respectively. The large median difference in the industrial wastewater loading could be explained by the possible underestimation by the iMFA, as mentioned in the previous section. The normalized median difference was particularly large for phosphorus (+ 2.44). The livestock feeding industry dominantly contributed to the phosphorus loading of industrial wastewater (Table S4), derived from the difference in wastewater generation for the livestock feeding industry between the iMFA and sMFA, as both models used the same phosphorus concentration data of the manure (Table S2). Because of the large variability, no expert could provide estimation on industrial wastewater generation. The PDFs (triangular) of industrial wastewater amount for each industry in the sMFA were based on two references [21, 22], whereas the PDFs (normal) in the iMFA were based on wide-ranging results of 65 industry surveys conducted in the present study (Table S4). These differences in the data source and created PDFs resulted in a smaller variability in industrial wastewater flow estimates in the sMFA than in the iMFA. As this flow accounted for 11% and 19%, respectively, for the total loading of nitrogen and phosphorus to the environment, the negative values [− 0.75 (Fig. 5) and − 0.70 (Figure S4)] of the normalized width of 80% CI of the industrial wastewater loading adversely affected the normalized width of 80% CI for the total loading to the environment (− 0.05 and − 0.11, respectively), indicating smaller variability in the estimates of total loading in the sMFA.

Apart from the four major flows, the large difference between the iMFA and sMFA was also observed in human excreta and fecal sludge, showing the limited performance of the sMFA. The normalized median difference for human excreta and fecal sludge was, respectively, + 1.00 and − 0.88 for nitrogen (Fig. 5) and + 1.00 and − 0.82 for phosphorus (Figure S4); the normalized widths of 80% CI were + 1.11 and − 0.88 for nitrogen and + 1.04 and − 1.03 for phosphorus. The median differences in human excreta corresponded to the proportions of the population without toilet facilities estimated by IS (median: 0.02) and LEJ (0.065), as shown in Fig. 2. This overestimation of LEJ could be explained by less-updated information by local experts under the rapidly increasing coverage of the septic tanks in urban areas of Mandalay region: 28 thousand in 2014 [32] to 330 thousand in 2019 [25]. The loading of fecal sludge was underestimated in sMFA compared with the iMFA, as shown in Fig. 5. This difference stems from IS and LEJ estimation differences on the proportion of fecal sludge discharged to the environment in Fig. 2c: 0.80 in IS, and 0.18 in LEJ, possibly caused by the active informal fecal sludge collecting business [15] not mentioned by the local experts. However, human excreta and fecal sludge were minor in both the iMFA and sMFA and did not significantly impact the sMFA overall.

### Sensitivity analysis for the parameters used in the sMFA development

The seven most sensitive parameters for the total pollution loading to the environment in the sMFA are summarized in Table 1. The unit loading in human excreta (C_2(N,P)_) was the highest for p10:p50 for nitrogen (ratio: 0.80) and p90:p50 for phosphorus (1.54), and the unit loading in greywater (C_3(N,P)_) was p90:p50 for nitrogen (1.35) and p10:p50 for phosphorus (0.81). The proportion of on-site sanitation effluent/leakage going to the environment (β_2,9_) was the second highest for p10:p50 and p90:p50 for nitrogen (0.84 and 1.39) and p10:p:50 for phosphorus (0.82). Among the 47 parameters based on PDFs, sensitivity analysis highlighted the importance of the unit pollution loadings in human excreta and greywater and the proportion of on-site sanitation effluent/leakage to the environment. Such findings are in line with those reported in the previous studies conducting sensitivity analysis for MFA [10, 11, 18], wherein the unit pollution loadings in human excreta and/or greywater were the most sensitive parameters for nutrient flow in each study area.

**Table 1:** Sensitivity analysis for the parameters in simplified material flow analysis (sMFA) to the total nitrogen and phosphorus loading to the soil and water environment

Parameter	Nitrogen		Phosphorus	
	Ratio	Rank	Ratio	Rank
Unit loading in human excreta (g/cap/day)				
p10:p50	0.80	1	0.88	3
p90:p50	1.31	3	1.35	1
Unit loading in greywater (g/cap/day)				
p10:p50	0.84	3	0.81	1
p90:p50	1.54	1	1.30	2
Proportion of on-site sanitation effluent/leakage going to the soil and water environment (unitless)				
p10:p50	0.84	2	0.82	2
p90:p50	1.39	2	1.29	3
Proportion of industrial solid waste going to the soil and water environment (unitless)				
p10:p50	0.96	4	0.97	7
p90:p50	1.06	5	1.05	4
Amount of industrial solid waste generation (ton/year)				
p10:p50	0.97	5	0.97	8
p90:p50	1.03	6	1.02	10
Amount of industrial wastewater generation (m^3^/year)				
p10:p50	0.97	5	0.95	5
p90:p50	1.03	6	1.04	5
Proportion of greywater going to the soil and water environment (unitless)				
p10:p50	0.98	8	0.96	4
p90:p50	1.00	11	1.00	8

The sensitivity was evaluated by the ratio of the total loading with the 10%tile or 90%tile values of a tested parameter and the 50%tile values of other parameters to that of the 50%tile values of all the parameters (p10:p50 or p90:p50). The table summarizes the five most sensitive parameters for p10:p50 and p90:p50 by nitrogen and phosphorus

Although the parameter variability of the unit loading in human excreta and the proportion of on-site sanitation effluent/leakage were relatively small (Table S2), there was high sensitivity to the total environmental load. This sensitivity is due to the loading of effluent/leakage from on-site sanitation, which received human excreta, was large, accounting for 57% and 46% of the total load to the environment for nitrogen and phosphorus, respectively (Figure S3). In contrast, the parameter variability of the unit loading in greywater was large (Table S2), and the loading of greywater to the environment was also high, accounting for 22% and 23% of the total loading for nitrogen and phosphorus, respectively (Figure S3). Therefore, the high sensitivity of the unit loading in greywater to the total environmental load was caused by the variability of the parameter itself and the large size of the flow relevant to greywater. The present study could not find the local data of the unit loading in greywater in Myanmar, and therefore used the data from the previous studies in other Southeast Asian countries (*n* = 12 for nitrogen and *n* = 8 for phosphorus) (Table S2), potentially increasing the uncertainty of the parameter. Although LEJ could complement local statistics on waste and wastewater management and provide good estimation in the MFA, supplementary surveys on sensitive and largely variable parameters, such as unit loading in greywater, would increase the accuracy of the sMFA. Such a focused and limited-resource survey would effectively decrease the uncertainty and increase the accuracy of the material flow.

## Conclusions

The present study aimed to examine the impact of the simplification on the uncertainty of the sMFA employing LEJ and to validate the effectiveness of the sMFA. Stochastic models of sMFA and iMFA were developed for nitrogen and phosphorus in urban Mandalay, Myanmar, and they were compared each other. The study showed that sMFA and iMFA estimated similar total loadings of nitrogen and phosphorus to the environment estimated, demonstrating the limited impact of the simplification of the MFA by employing LEJ on the uncertainty and the effectiveness of the sMFA. This indicates the potentials of LFJ and sMFA to complement iMFA to achieve the effective understanding of material flows for urban waste and wastewater management, especially in low- and middle-income settings, where secondary data are often limited.

However, there are some limitations to the sMFA. Compared with the iMFA, the sMFA showed gaps in nitrogen and phosphorus loading estimation of the flows related to informal activities: fecal sludge, industrial wastewater, and industrial solid waste management. The LEJ may not work well for quantitatively estimating informal activity. If the flows related to informal activities are major in a city, additional investigations on the activities are required to strengthen the sMFA. Another limitation is the lack of unit loading data for waste and wastewater: human excreta and greywater in this study. Although the sensitivity of these parameters was high in this study, it was difficult to estimate these parameters by local experts. Along with the use of LEJ, an on-the-ground investigation of fundamental unit loading data would increase the accuracy of the sMFA. Furthermore, the present study did not investigate the impact of selecting local experts, potentially affecting the estimation of the sMFA. Nevertheless, this study showed the potential usefulness of the sMFA that will complement the iMFA, especially under data-limited conditions.

## Supplementary Information

Below is the link to the electronic supplementary material.

Supplementary file1 (PDF 1727 KB)
