# Supplementary material for: A simplified material flow analysis employing local expert judgment and its impact on uncertainty
Source: J Mater Cycles Waste Manag. 2023 May 2;25(4):2101–12. doi: 10.1007/s10163-023-01660-5 (PMC10307724; doi:10.1007/s10163-023-01660-5)
Supplement: Supplementary file 1 — Supplementary file1 (PDF 1727 KB) [file 10163_2023_1660_MOESM1_ESM.pdf]

Supporting Information

A simplified material flow analysis employing local expert judgment and its impact on uncertainty

Wutyi Naing, Hidenori Harada*, Shigeo Fujii, Chaw Su Su Hmwe

Corresponding to: Hidenori Harada, Graduate School of Asian and African Area Studies, Kyoto University, 46 Yoshida-shimoadachi-cho, Sakyo, Kyoto 606-8501, Japan
E-mail: harada.hidenori.8v@kyoto-u.ac.jp

Table S1. Reaction process matrix of the MFA

**Component [X_j]**		**Transfer factor to component *j* through process *i* [$\beta_{i,j}$] (unitless)**												**Reaction rate of process *i* [ρ_i] (tons/year)**
**Reaction flow process [P_i] ↓**		X_1	X_2	X_3	X_4	X_5	X_6	X_7	X_8	X_9	X_{10}	X_{11}	X_{12}	
P_1	Market food for human	1					-1							$P_2 + P_3 + P_4 - P_5 - (P_8 \times \beta_{8,1})$
P_2	Human excreta	-1	$\beta_{2,2}$							$\beta_{2,9}$				$C_{2,N(P)} \times P_0 \times 356 \times 10^{-6}$
P_3	Greywater	-1	$\beta_{3,2}$							$\beta_{3,9}$				$C_{3,N(P)} \times P_0 \times 356 \times 10^{-6}$
P_4	Food waste from household	-1		$\beta_{4,3}$					$\beta_{4,8}$	$\beta_{4,9}$				$C_{4,N(P)} \times P_0 \times 356 \times 10^{-6}$
P_5	Water consumption by households	1								-1				$((C_{5gw,N(P)} \times Q_5 \times \alpha_{gw}) + (C_{5sw,N(P)} \times Q_5 \times \alpha_{sw})) \times P_0 \times 356 \times 10^{-6}$
$P_{6\text{-iMFA}}$	Fecal sludge from on-site sanitation		-1					$\beta_{6,7}$		$\beta_{6,9}$				$F_{empty-survey} \times N_{os} \times Q_{fs} \times C_{6,N(P)} \times 10^{-6}$
$P_{6\text{-sMFA}}$														$(V_{os} \times N_{os} \times \alpha_{fs-st})/F_{empty-refer}$
P_7	Toilet effluent/leakage		-1							1				$P_2(\beta_{2,2}) + P_3(\beta_{3,2}) - P_6$
P_8	Animal products	$\beta_{8,1}$		-1			$\beta_{8,6}$							$C_{8,N(P)(pig+cattle+poultry)} \times W_{8(pig+cattle+poultry)}) + (C_{8N(P)mi} \times Q_8)$
P_9	Animal feeding			1			-1							$P_8 + P_{10} - P_{4(\beta_{4,3})} - P_{11} - P_{14(\beta_{14,3})} - P_{21(\beta_{21,3})} - P_{24}$
P_{10}	Manure			-1						$\beta_{10,9}$			$\beta_{10,12}$	$C_{10,N(P)} \times N_{10} \times 365 \times 10^{-6}{}_{\text{(pig+cattle+poultry)}}$
P_{11}	Water consumption by livestock			1						-1				$(C_{5gw,N(P)} \times Q_{11}) \times 365 \times 10^{-6}$
P_{12}	N fixation from paddy field				1						-1			$F_{12} \times S_{agri}$
P_{13}	Agriculture products				-1		1							$F_{12} \times S_{agri}$
P_{14}	Residue from agriculture			$\beta_{14,3}$	$-1+\beta_{14,4}$									$P_{13} \times \alpha_{resi}$
P_{15}	Water consumption by agriculture				1					-1				$C_{15,N(P)} \times Q_{agri} \times S_{agri} \times 10^{-4}$
P_{16}	Fertilizer demand				1		-1							$Q_{16} \times S_{agri} \times C_{16,N(P)} \times 10^3$
P_{17}	N emission from agriculture				-1						1			$P_{16} \times k_{N,cf}$

P_{18}	Agriculture runoff				-1					1			$(P_{16} + P_{12} + P_{14(\beta_{14,4})} + P_{15}) \times ro_{N(P)}$
P_{19}	Production from industry					-1	1						$C_{19,N(P)} \times Q_{19}$
P_{20}	Industrial wastewater					-1				1			$C_{20,N(P)} \times Q_{20} \times 10^{-6}$
P_{21}	Solid waste from industry			$\beta_{21,3}$		-1			$\beta_{21,8}$	$\beta_{21,9}$		$\beta_{21,11}$	$C_{21,N(P)} \times Q_{21}$
P_{22}	Water consumption by industry					1				-1			$C_{5gw,N(P)} \times Q_{22} \times 10^{-6}$
P_{23}	Raw material for industry					1	-1						$P_{19} + P_{20} + P_{21} - P_{22}$
P_{24}	Market waste for animal feeding			1			-1						$Q_{24} \times R_{24}$

- Explanation on how to read Table S1 based on Eq. (1) and (2).

For an example of P_2 (human excreta), the amount of human excreta generated in the study area is calculated by the reaction rate based on unit N or P generation per capita per day and population data. All of human excreta will output from household (X_1) to somewhere. Therefore, the transfer factor of P_2 at X_1 is "-1". Then, a proportion ($\beta_{2,2}$) of P_2 (human excreta) is input into onsite sanitation (X_2). The remaining ($\beta_{2,9}$) is input into X_9 (Soil and water environment), meaning direct discharge of human excreta to water channel. It explains as all of human excreta is generated at and outputted from X_1 (household), and then transferred to either of X_2 (Onsite sanitation) or X_9 (Soil and water environment). Considering the mass balance law, the sum of "-1", "$\beta_{2,2}$" and "$\beta_{2,9}$" should be zero. Accordingly, Eq. (2) for P_2 is zero.

In real field, phosphorus could be accumulated in the soil of farmland. In this MFA, the component where phosphorus is accumulated (X_9, i.e. soil and water environment) is out of the system boundary as shown in Figure S1. For example, P_{18} indicates the flow from agriculture to X_9, which includes phosphorus accumulation amount per unit time into soil as well as runoff to water environment. It means phosphorus accumulation is not included in "Agriculture" within the system boundary but included in soil out of the system boundary. Accordingly, as far as equation (1) is applied to components within system boundary, Eq. (1) will be 0, meaning no accumulation at the components within the system boundary. Similarly, X_6 (Market) and X_{10} (Atmosphere) are geographically located inside the target area but conceptually excluded from the inner boundary to simplify mass balance of the components inside the system boundary (Figure S1). In the model, the market is the origin of goods to Household, chemical fertilizer to Agriculture, organic solid waste to Livestock, commercial feed to Livestock, and raw materials to Industry; also it is the destination of products from Agriculture, products from Livestock, and products from Industry.

Table S2. Secondary data for the reaction rates of reaction processes

Process	Explanation and Symbol	Unit	Value (min-med-max)	*n*	Distribution type	Reference
**Household** (X_1)	Total population (P_0)	person	1,225,546	-	-	1
Human excreta (P_2)	N amount in human excreta (C_{2N})	g/cap/d	3.9-8.9-13.7	29	lognormal	2-10
	P amount in human excreta (C_{2P})	g/cap/d	0.7-1.4-4.1	21	lognormal	3-11
Greywater (P_3)	N amount in greywater (C_{3N})	g/cap/d	0.6-4.4-8.5	12	lognormal	12-18
	P amount in greywater (C_{3P})	g/cap/d	0.2-0.5-1.9	8	lognormal	11-16
Kitchen waste (P_4)	N amount in kitchen waste (C_{4N})	g/cap/d	0.5-0.7-0.9	6	lognormal	9,12,14,19
	P amount in kitchen waste (C_{4P})	g/cap/d	0.1-0.3-0.8	8	lognormal	11-14
Water consumption (P_5)	N concentration in ground water (C_{5Ngw})	mg/l	6.2-8.9-13.1	10	lognormal	20
	N concentration in surface water (C_{5Nsw})	mg/l	8.3-9.7-13.1	4	triangular	20
	P concentration in ground water (C_{5Pgw})	mg/l	0.01-0.05-0.07	10	lognormal	20
	P concentration in surface water (C_{5Psw})	mg/l	0.01-0.03-0.05	4	triangular	20
**On-site sanitation** (X_2)						
Fecal sludge (P_6)	N concentration in fecal sludge (C_{6N})	mg/l	245.0-459.5-627.0	4	triangular	21, 22, 23
	P concentration in fecal sludge (C_{6P})	mg/l	150.0-191.2-250.0	4	triangular	21, 22
	Amount of fecal sludge collected per trip (Q_{fs})	m^3	4.5	-	-	24
	Total trips transported to disposal pond (Q_{trip})	trip/year	3,690	-	-	24
	Total on-site unit in the study area (N_{os})	Unit	213,104	-	-	25
	Average volume of onsite sanitation	m^3	4.5	-	-	24
	Desludging frequency ($F_{empty\text{-}refer}$)	year/time	6.2-9.2-14.2	4	triangular	17, 26, 27
	Proportion of each septic tank which is faecal sludge (i.e. not effluent, supernatant or infiltrate) ($\alpha_{fs\text{-}st}$)	unitless	0.5	-	-	27
Toilet effluent (P_7)	Ratio of N accumulation in septic tank ($\alpha_{ac(N)sep}$)	unitless	0.05-0.09-0.10	3	triangular	9
	Ratio of P accumulation in septic tank ($\alpha_{ac(P)sep}$)	unitless	0.11-0.19-0.27	3	triangular	9
	Ratio of N accumulation in pit latrine ($\alpha_{ac(N)pit}$)	unitless	0.17-0.18-0.27	3	triangular	9
	Ratio of P accumulation in pit latrine ($\alpha_{ac(P)pit}$)	unitless	0.18-0.29-0.40	3	triangular	9
**Livestock** (X_3)						
Animal production(P_8)	N amount in milk (C_{8Nmi})	g/kg	5.4-4.9-4.4	1*	normal	13
	P amount in milk (C_{8Pmi})	g/kg	1.1-1.0-0.9	1	normal	13
	N amount in meat (pig) (C_{8Npi})	g/kg	27-26-25	1	normal	13
	N amount in meat (cattle) (C_{8Nca})	g/kg	28.5-27.5-26.5	1	normal	13

	N amount in meat (poultry) (C_{8Npo})	g/kg	31-32-33	1	normal	13
	P amount in meat (pig) (C_{8Ppi})	g/kg	1.55-1.50-1.45	1	normal	13
	P amount in meat (cattle) (C_{8Pca})	g/kg	1.75-1.7-0.65	1	normal	13
	P amount in meat (Poultry) (C_{8Ppo})	g/kg	2.2-2.0-1.8	1	normal	13
	Amount of milk production (Q_8)	ton/year	1960		-	1
	Amount of meat production (pig) (W_{8pi})	ton/year	3295		-	1
	Amount of meat production (cattle) (W_{8ca})	ton/year	391		-	1
	Amount of meat production (poultry) (W_{8po})	ton/year	11952		-	1
Livestock manure (P_{10})	N amount in manure (pig) (C_{10Npi})	g/head/d	17.5-20.4-23.4	3	triangular	22, 28
	N amount in manure (cattle) (C_{10Nca})	g/head/d	31.7-128.9-198.0	7	lognormal	22, 28, 29, 30
	N amount in manure (poultry) (C_{10Npo})	g/head/d	0.4-1.2-1.6	4	triangular	22, 28, 30
	P amount in manure (pig) (C_{10Ppi})	g/head/d	4.6-6.8-8.1	3	triangular	22, 28
	P amount in manure (cattle) (C_{10Pca})	g/head/d	5.1-28.3-34.8	6	lognormal	22, 28, 29
	P amount in manure (poultry) (C_{10Ppo})	g/head/d	0.08-0.6-1.2	3	triangular	22, 28
	Total number of livestock (pig) (N_{10pi})	head	9,952		-	1
	Total number of livestock (cattle) (N_{10ca})	head	6,626		-	1
	Total number of livestock (poultry) (N_{10po})	head	318,176		-	1
Water consumption (P_{11})	Amount of water consumption (Q_{11})	m^3/day	361-691-1677	3	triangular	31,32
**Agriculture (X4)**						
Nitrogen fixation (P_{12})	Nitrogen fixation from paddy field (F_{12})	kg/ha	9-100-149	13	normal	13, 33, 34, 35, 36
Agricultural production (P_{13})	Agricultural land area (S_{agri})	km^2	0.8		-	1
	Total rice field area ($S_{agri(rice)}$)	km^2	0.5		-	1
	Total vegetable field area ($S_{agri(veg)}$)	km^2	0.3		-	1
	Amount of agricultural production (rice) ($P_{agri(rice)}$)	ton/year	330		-	1
	Amount of agricultural production (veg) ($P_{agri(veg)}$)	ton/year	180		-	1
Agricultural residue (P_{14})	N amount in agricultural product (rice) (C_{13Nri})	g/kg	10.7-11.4-13.4	4	triangle	13, 15, 37
	N amount in agricultural product (veg) (C_{13Nveg})	g/kg	4.7-5.6-6.7	4	triangle	15, 38, 39
	P amount in agricultural product (rice) (C_{13Pri})	g/kg	1.3-2.4-3.8	4	triangle	13, 15
	P amount in agricultural product (veg) (C_{13Pveg})	g/kg	0.40-0.48-0.55	4	triangle	13, 39
	Ratio of agricultural residue to product (rice) ($\alpha_{res(rice)}$)	unitless	0.53-0.59-0.64	5	triangle	13, 35, 40, 41, 42

	Ratio of agricultural residue to product (veg) ($\alpha_{res(veg)}$)	-	0.37-0.53-0.75	6	normal	35, 40, 41
Water consumption (P_{15})	Agricultural water consumption (Q_{agri})	m^3/ha	14015-16200	2	triangle	15, 43
	N concentration in irrigation water (C_{15N})	mg/l	0.3-2.1-6.9	9	lognormal	44
	P concentration in irrigation water (C_{15P})	mg/l	0.3-2.0-9.3	9	lognormal	44
N emission (P_{17})	N emission factor for paddy field (k_N)	unitless	0.06-0.13-0.16	4	triangle	38, 39
Agricultural runoff (P_{18})	Ratio of N runoff (ro_N)	unitless	0.01-0.25-0.3	6	normal	23, 29, 45, 46, 47
	Ratio of P runoff (ro_P)	unitless	0.01-0.02-0.04	8	normal	23, 45, 46
**Industry** (X_5)						
Industrial production (P_{19})	Amount of industrial production (Q_{19})	ton/year	313282		normal	48
Industrial solid waste (P_{21})	N proportion in organic solid waste (C_{21N})	%	2.0-2.7-3.8	5	triangular	19, 49, 50, 51
	P proportion in organic solid waste (C_{21P})	%	0.4-0.43-0.5	4	triangular	19, 50, 51
**Market** (X_6)						
Market Waste for animal feeding(P_{24})	Amount of organic market waste generated (Q_{24})	ton/day	50-51-52	36	normal	52
	Ratio of livestock feeding from market vegetable waste (R_{24})	unitless	0.05	-	beta	52

*one data with standard deviation.

Table S3. Primary data for stochastics iMFA and sMFA with distribution

Process	Item (symbol)	Intensive survey n	Intensive survey Ratio	Local expert judgement n	Local expert judgement Ratio	Distribution type
**Household** (X_1)						
Human excreta (P_2)	Ratio of human excreta going to onsite sanitation ($\beta_{2,2}$)	400	0.98	10	0.935	beta(n, α, β)[a]
	Ratio of human excreta going to ground soil/surface water/drainage ($\beta_{2,9}$)	400	0.02	10	0.065	beta(n, α, β)
Greywater (P_3)	Ratio of grey water going to onsite sanitation ($\beta_{3,2}$)	400	0.01	10	0.03	beta(n, α, β)
	Ratio of greywater going to ground soil/surface water/drainage ($\beta_{3,9}$)	400	0.99	10	0.97	beta(n, α, β)
Kitchen waste (P_4)	Ratio of kitchen waste going to livestock ($\beta_{4,3}$)	400	0.01	10	0.17	beta(n, α, β)
	Ratio of kitchen waste going to ground soil/surface water/drainage ($\beta_{4,9}$)	400	0.19	10	0.18	beta(n, α, β)
	Ratio of kitchen waste going to disposal sites ($\beta_{4,8}$)		0.80[57)]	10	0.65	beta(n, α, β)
Water consumption (P_5)	Ratio of surface water consumption (α_{sw})	400	0.05	10	0.10	beta(n, α, β)
	Ratio of groundwater consumption by household (α_{gw})	400	0.95	10	0.90	beta(n, α, β)
**On-site sanitation** (X_2)						
Fecal sludge (P_6)	Ratio of fecal sludge going to disposal ponds ($\beta_{6,7}$)	400	0.20	10	0.82	beta(n, α, β)
	Ratio of fecal sludge going to ground soil/surface water/drainage ($\beta_{6,9}$)	400	0.80	10	0.18	beta(n, α, β)
Toilet effluent (P_7)	Ratio of septic tank (-)	400	0.84	10	0.80	beta(n, α, β)
	Ratio of pit latrine (-)	400	0.16	10	0.20	beta(n, α, β)
**Livestock** (X_3)						
Livestock production (P_8)	Ratio of animal product going to household ($\beta_{8,1}$)	21	0.32	9	0.17	beta(n, α, β)
	Ratio of animal product going to market ($\beta_{8,6}$)	21	0.68	9	0.83	beta(n, α, β)
Livestock manure (P_{10})	Ratio of manure going to agricultural activities outside the system boundary ($\beta_{10,12}$)	21	0.57	9	0.58	beta(n, α, β)
	Ratio of manure going to ground soil/surface water/drainage ($\beta_{10,9}$)	21	0.43	9	0.42	beta(n, α, β)
**Agriculture (X4)**						
Agricultural residue (P_{14})	Ratio of residue going to livestock feeding (rice) ($\beta_{14,3(rice)}$)	10	0	10	0.47*	beta(n, α, β)
	Ratio of residue going to livestock feeding (veg) ($\beta_{14,3(veg)}$)	10	0.16			beta(n, α, β)
	Ratio of residue going to agriculture (rice) ($\beta_{14,4(rice)}$)	10	1.00	10	0.53*	beta(n, α, β)
	Ratio of residue going to agriculture (veg) ($\beta_{14,4(veg)}$)	10	0.84			beta(n, α, β)
**Industry** (X_5)						
Industrial solid waste (P_{21})	Ratio of industrial solid waste used for livestock feeding activities inside the system boundary ($\beta_{21,3}$)	65	0.11	10	0.11	beta(n, α, β)
	Ratio of industrial solid waste used for livestock feeding activities outside the system boundary ($\beta_{21,11}$)	65	0.72	10	0	beta(n, α, β)
	Ratio of industrial solid waste going to disposal sites ($\beta_{21,8}$)	65	0.11	10	0.73	beta(n, α, β)
	Ratio of industrial solid waste going to soil/surface water ($\beta_{21,9}$)	65	0.06	10	0.16	beta(n, α, β)

[a]n= number of integration, $\alpha = \mu\nu$, $\beta = (1 - \mu)\nu$, ν=sample number

*Estimation of the ratios of agricultural residue going to livestock feeding and agriculture were integrated for rice and vegetable.

Table S4. Estimation of amount and standard deviation of industrial wastewater and solid waste

	Wastewater								Solid waste		
Model type	Intensive (iMFA)			Simplified (sMFA)			Both		Intensive (iMFA)		
Parameter	Amount (m^3/year)	SD	distribution type	Amount (m^3/year)	Amount (m^3/year)	Distribution type	TN(mg/l)	TP(mg/l)	Amount (ton/year)	SD	distribution
Type of industry	(IS)			(WHO 1993)	(OECC 2003)		(JSWA 1999)				
Oil_L	168630	101582	normal	12328	18054	triangular	20	29	13996	18692	normal
Beverage_L	278404	167710	normal	1993998	352920	triangular	172	8	5781	7720	normal
Softdrink_L	42924	25857	normal	51200	39290	triangular	19	3	283	378	normal
Coffeemix_L	9928	5981	normal	17820	46775	triangular	33	4	222	296	normal
Tealeaf_L	5953	3586	normal	22586	7358	triangular	49	19	55	74	normal
Sugar_L	2920	1759	normal	299472	2015821	triangular	37	2	5793	7736	normal
Candy_L	5786254	3485623	normal	57816	313170	triangular	56	9	100	133	normal
Rusk_L	148920	89709	normal	259682	750191	triangular	56	9	828	1106	normal
Noodle_L	14162	8531	normal	446	324	triangular	22	8	10	13	normal
Flour_L	111690	67282	normal	793386	1009836	triangular	12	3	1146	1531	normal
Livestockfeed_L	14965	9015	normal	4087866	1824940	triangular	126	66	14	18	normal
Milling_L*	0	-	normal	17043	567791	triangular	0	0	32851	43872	normal
PreservedMeat_L	11169	6728	normal	2500	1800	triangular	101	21	1	2	normal
Icecream_L	19345	11653	normal	113	488	triangular	35	9	331	442	normal
Oil_M	0	-	normal	2135	3138	triangular	0	0	2060	2751	normal
Icecream_M	19345	11653	normal	113	488	triangular	35	9	331	442	normal
Rusk_M	15476	9323	normal	5256	15184	triangular	56	9	69	92	normal
Flour_M	3285	1979	normal	80399	102333	triangular	12	3	464	619	normal
Milling_M*	0	-	normal	350	11644	triangular	0	0	7616	10172	normal
Sauce_M	6935	4178	normal	11	27	triangular	49	8	147	196	normal
C.Milk_M	876	527.7	normal	803	1205	triangular	35	9	0.1	0.5	normal
Oil_S	657	396	normal	7	11	triangular	20	29	33	44	normal

Sugar_S	1113	671	normal	3024	20355	triangular	37	2	508	678	normal
Rusk_S	17885	10774	normal	1314	3796	triangular	56	9	5	7	normal
Flour_S	657	396	normal	5346	6804	triangular	12	3	151	201	normal
Milling_S*	0	-	normal	287	9545	triangular	0	0	610	814	normal

iMFA means intensive material flow analysis and sMFA means simplified material flow analysis. IS means intensive survey. SD means standard deviation. L, M and S mean large, median and small, respectively, for the sizes of industries.

Sizes of industries were distinguished by the number of labors; 15-50 labors for the small-scale, 51-300 labors for the median-scale and, 301 and more labors for the large-scale industries (MOI 2016).

*TN and TP in the wastewater were referred from WHO 1993 because JSWA 1999 did not mention them.

Table S5. Number, type and size of food and beverage factories in urban Mandalay

Type	**No. of factories by size in the study area**				**Surveed no. of factories**			
	**Large**	**Median**	**Small**	**Total**	**Large**	**Median**	**Small**	**Total**
Oil	21	33	1	55	3	1	1	
Beverage	3			3	4			
Soft drink	4			4	3			
Coffee mix	1			1	1			
Ice cream	1	1		2	1			
Tealeaf	1			1			1	
Sugar	2		1	3	2	2		
Jelly, candy	3			3	2	1		
Rusk	17	4	1	22	12	7	1	
Noddle	2			2	1	1	1	
Flour	4	5	1	10	2	1	1	
Milling	22	11	2	35		5	3	
Animal feed	5			5	1			
Yeast	1			1				
Preserved meat	1			1		2		
Sauce		1		1			4	
Condensed milk						1		
Total	88	55	6	149	32	21	12	65

Source: (48)

Text S1. Industrial wastewater generation estimation

Industrial wastewater generation was estimated by the following equation. The details are summarized in Table 3S.

$$Q_{20} = \sum_w (Pro_{i\,w} \times U_w) \qquad \text{Eq. (S1)}$$

where, Q_{20} = industrial wastewater generation (m^3/year),

Pro_{i_w} = industrial production of w type industry (ton/year)

U_w = wastewater generation from a unit production of w type industry (m^3/ton)

For iMFA, industrial water consumption, wastewater generation and solid waste generation were estimated from 65 industrial managers from different types of industries. This study assumed that the shape of probability distribution was parallelly the same for every type and size of industry and the size of distribution was parallelly moved. Therefore, standard deviation (SD) for industrial water consumption, wastewater and solid waste generation for each industry was estimated proportionally from the SD of Rusk (large size) industry which had 12 sample numbers, the largest sample number among different types of industries in 65 industrial manager interview survey (Table 4S). Normal distribution with estimated SD was used for industrial water consumption, wastewater generation and solid waste generation in iMFA.

For sMFA, Industrial wastewater generation, as mentioned before, was estimated based on two different reports and triangular distribution was used for each type of industry. Solid waste generation was estimated by the 9 out of 10 local experts who participated in our survey, not distinguishing between the types of industries, but integrating as an overall solid waste generation (ton/year) from all of the industries, and the normal distribution was used. The parameters such as mean and standard deviation (SD) to get PDF were defined from the fitdisplus package in R version 3.6.1.

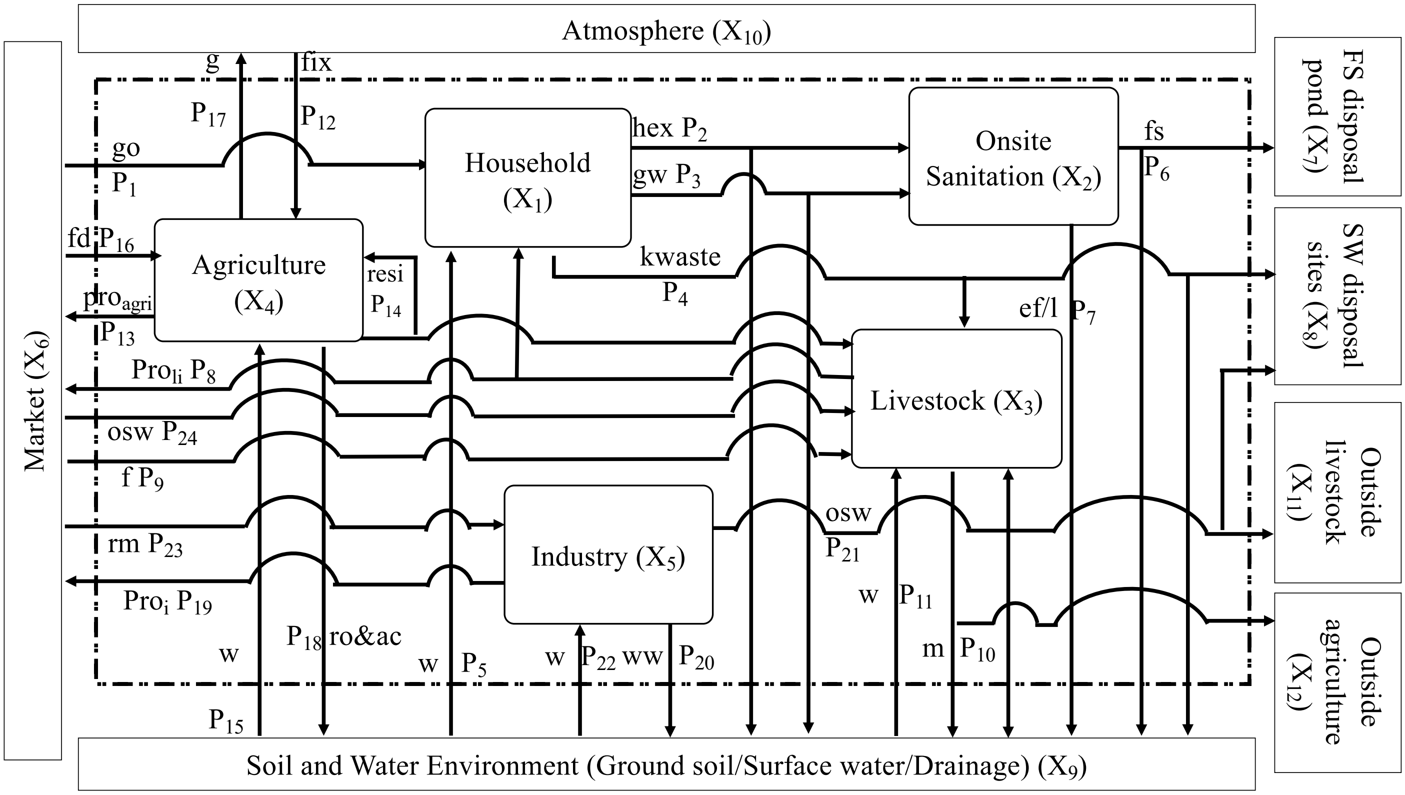

Figure S1. A schematic diagram of a material flow model. P*n* indicate each reaction flow process. go= goods, hex = human excreta, gw = greywater, kwaste = kitchen waste, w = water, fs = fecal sludge, ef/l = effluent/leakage, pro_n = production of a component, f = feeding, m = manure, fix = N fixation, resi = residue, fd = chemical fertilizer demand, g = gas, ro&ac = runoff and soil accumulation, ww = wastewater, osw= organic solid waste, rm = raw material

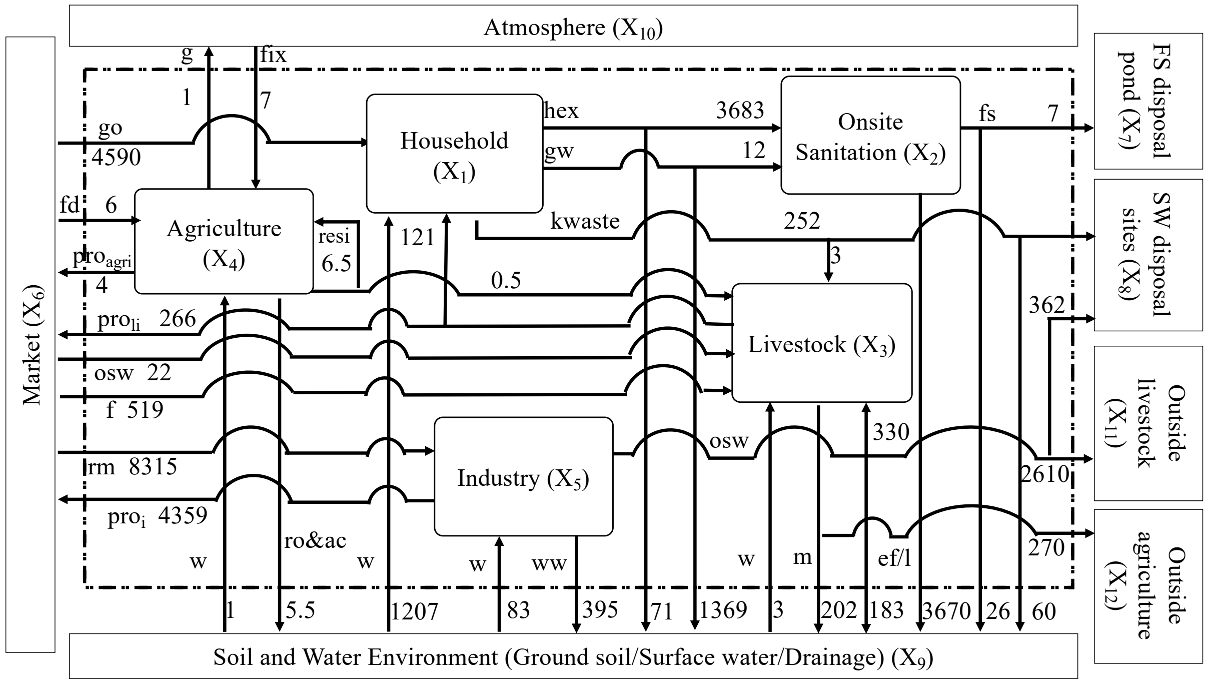

(a). iMFA nitrogen flow (ton-N/year).

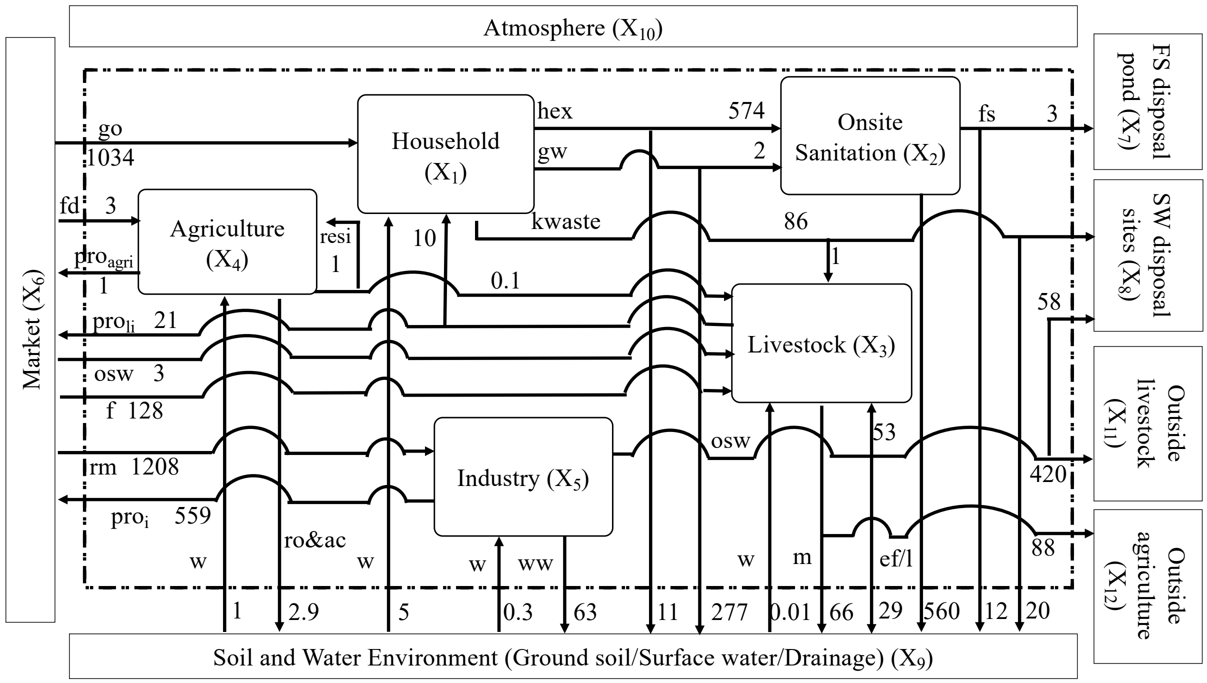

(b). iMFA phosphorus flow (ton-P/year).

Figure S2. Intensive MFA (iMFA) estimation of nitrogen and phosphorus flows as median values of the stochastic modeling. Abbreviation indicate as follows: go= goods, hex = human excreta, gw = greywater, kwaste = kitchen waste, w = water, fs = fecal sludge, ef/l = effluent/leakage, pro_n = production of a component, f = feeding, m = manure, fix = N fixation, resi = residue, fd = chemical fertilizer demand, g = gas, ro&ac = runoff and soil accumulation, ww = wastewater, osw= organic solid waste, rm = raw material. The total loading to Soil and Water Environment (X_9) is a sum of input from Household (X_1; hex, gw and kwaste), Onsite Sanitation (X_2; ef/l and fs), Livestock (X_3; m), Agriculture (X_4; ro&ac), Industry (X_5; ww and osw).

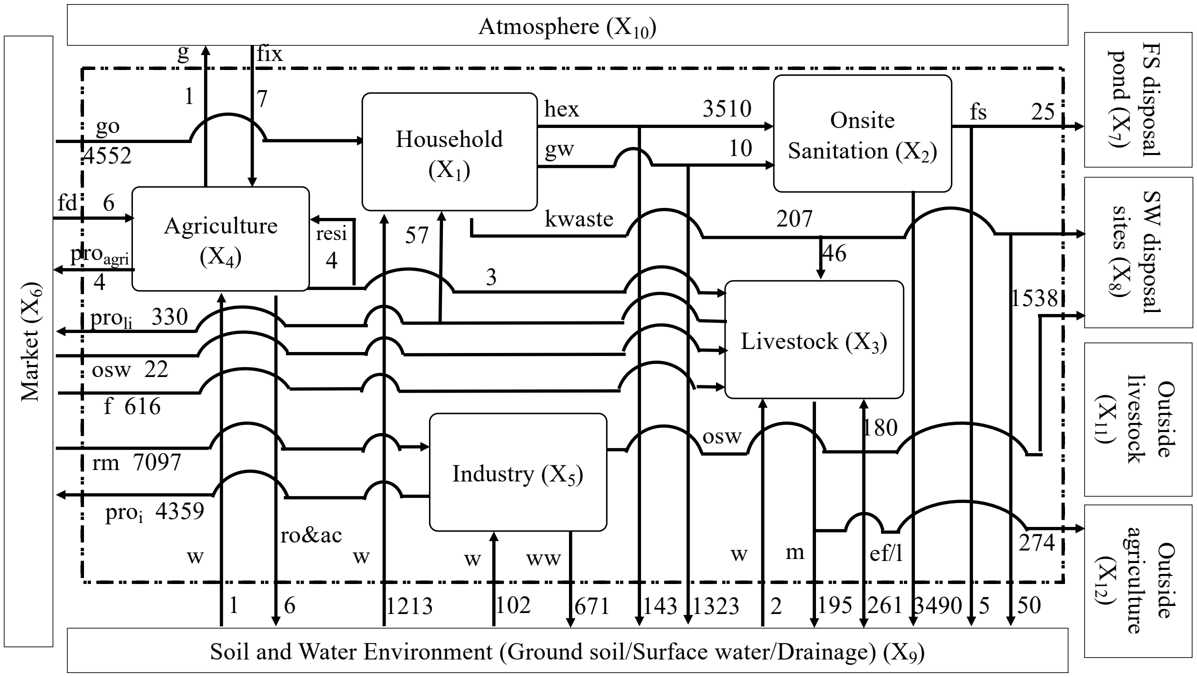

(a). sMFA nitrogen flow (ton-N/year).

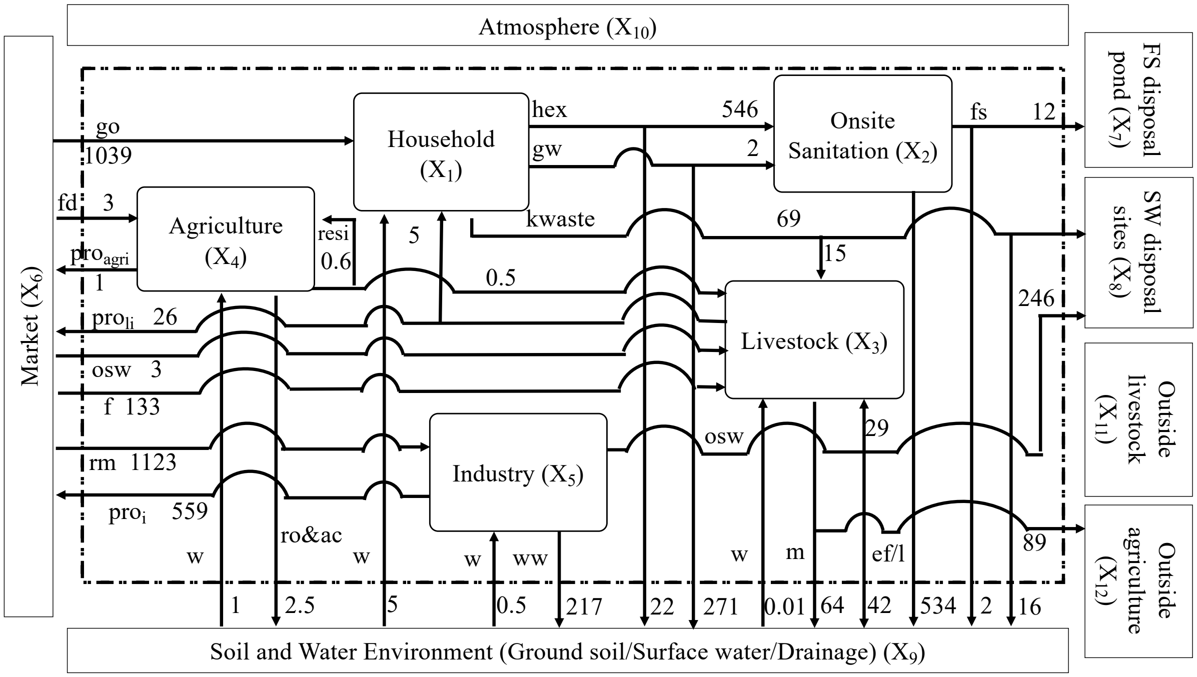

(b). sMFA phosphorus flow (ton-P/year).

Figure S3. Simplified MFA (sMFA) estimation of nitrogen and phosphorus flows as median values of the stochastic modeling. Abbreviations indicate as follows: go= goods, hex = human excreta, gw = greywater, kwaste = kitchen waste, w = water, fs = fecal sludge, ef/l = effluent/leakage, pro_n = production of a component, f = feeding, m = manure, fix = N fixation, resi = residue, fd = chemical fertilizer demand, g = gas, ro&ac = runoff and soil accumulation, ww = wastewater, osw= organic solid waste, rm = raw material. The total loading to Soil and Water Environment (X_9) is a sum of input from Household (X_1; hex, gw and kwaste), Onsite Sanitation (X_2; ef/l and fs), Livestock (X_3; m),Agriculture (X_4; ro&ac), Industry (X_5; ww and osw).

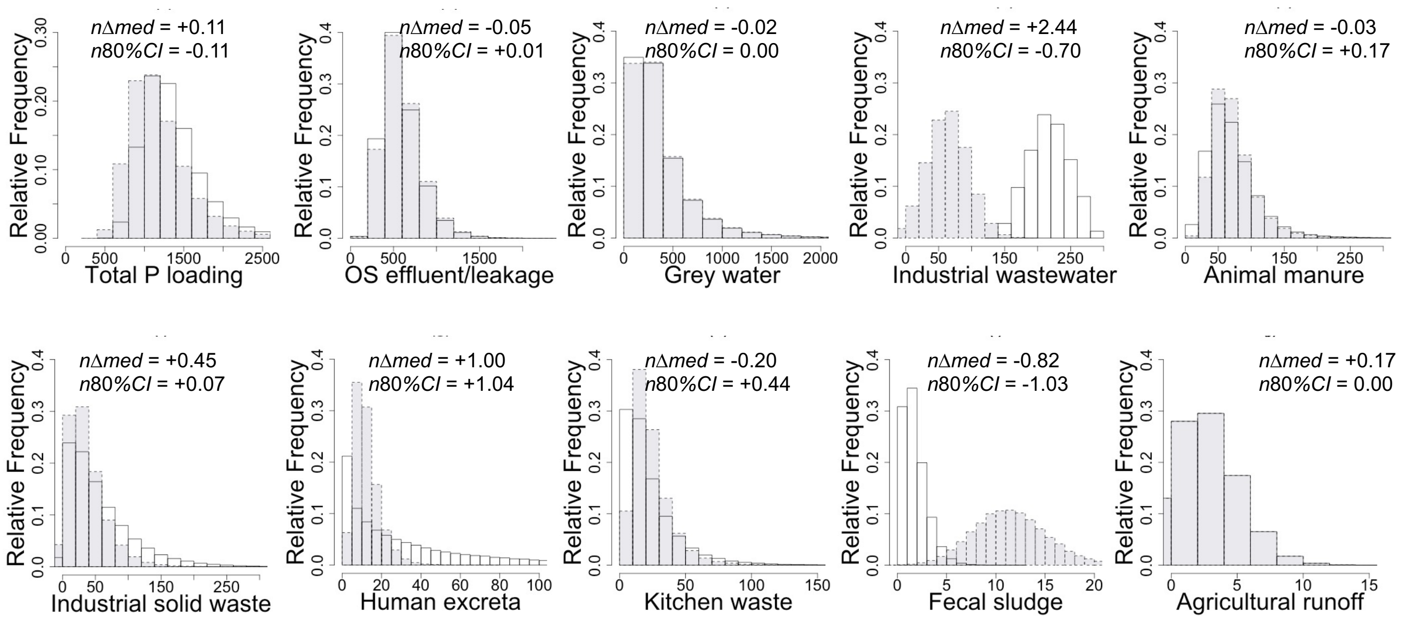

Figure S4. Histogram of phosphorus loadings to soil and water environment of the stochastic iMFA and sMFA with 100,000 iterations (tons/year). Grey histograms were referred to iMFA and white ones to sMFA. OS means onsite sanitation. *n∆med* means normalized median difference and *n80%CI* means normalized width of 80% confidence interval.

References

(1) GAD (General Administration Department). 2017. "Regional data report, Mandalay district, 2017." (October). GAD, Mandalay.

(2) Eawag/Sandec, 1998. Solids Separation and Pond Systems for the Treatment of Faecal Sludges in the Tropics 88.

(3) Cross, P. and Strauss, M., 1985. Health Aspects of Nightsoil and Sludge Use in Agriculture and Aquaculture. Part I: Existing Practices and Beliefs in the Utilization of Human Excreta. Part II: Pathogen Survival. IRCWD Report No. 04/85.

(4) Schouw, N.L., Danteravanich, S., Mosbaeck, H., Tjell, J.C., 2002. Composition of human excreta-a case study from Southern Thailand. Sci. Total Environ. 286, 155–166.

(5) Heinss U., Larmie S.A., Strauss M., 1998. Solid separation and pond systems for the treatment of faecal sludges in the tropics. SANDEC Report 5/98; EAWAG, Duebendorf, Switzerland.

(6) Jonsson H., Richert Stintzing, A., Vinnera˚ s, B., Salomon, E., 2004. Guidelines on the use of urine and faeces in crop production. Report 2004–2, EcoSanRes Publications Series, Stockholm Environment Institute.

(7) Polprasert, C, Lohani, B.N, Chan, C.B., 1981. Human faeces, urine and their utilization. V.A. Tuan, D.M. Tam (Trans.), Reviewer Feachem, R. ENSIC Translation Committee.

(8) Schouw, N.L., Tjell, J.C., Danteravanich, S., 2002. Availability and quality of solid waste and wastewater in Southern Thailand and its potential use as fertiliser 332–340.

(9) Montangero, A., Belevi, H., 2007. Assessing nutrient flows in septic tanks by eliciting expert judgement: a promising method in the context of developing countries. Water Res. 41, 1052–64. https://doi.org/10.1016/j.watres.2006.10.036

(10) Polprasert, C., 1996. Organic Waste Recycling. Technology and Management, second ed. Wiley.

(11) Tran, N. Q., 2016. Characterization of domestic wastewater discharge and its impact on material flows in urban Hue, Vietnam. PhD Thesis. Kyoto University.

(12) Kroiss, H., Rechberger, H., Egle, L., 2008. Phosphorus in Water Quality and Waste Management. Institute of Water Quality, Resources and Waste Management Vienna University of Technology Austria.

(13) Montangero, A., 2006, Material Flow Analysis for Environmental Sanitation Planning in Developing Countries, PhD Thesis. Leopold-Franzens-University Innsbruck.

(14) Strande, L., Ronteltap, M., Brdjanovic, D., 2014. Faecal Sludge Management. IWA Publishing. Chapter 2. Pg-24.

(15) Pham, H.G., Harada, H., Fujii, S., Nguyen, P.H.L., Huynh, T.H., 2016. Transition of human and livestock waste management in rural Hanoi: a material flow analysis of nitrogen and phosphorus during 1980–2010. J. Mater. Cycles Waste Manag. 1–13. https://doi.org/10.1007/s10163-016-0484-1

(16) Gold, M., Harada, H., Therrien, J.-D., Nishida, T., Cunningham, M., Semiyaga, S., Fujii, S., Dorea, C., Nguyen, V.-A., Strande, L., 2017. Cross-country analysis of faecal sludge dewatering. Environ. Technol. 0, 1–1. https://doi.org/10.1080/09593330.2017.1374472.

(17) Anh. Pham Nguyet., 2014. Study on household wastewater characterization and septic tanks' function in urban areas of Vietnam. PhD Thesis. Kyoto University. 10.1360/zd-2013-43-6-1064

(18) Nippon Koei Co., L. JICA Survey on Examination of Monitoring Methodology for Indicator of SDG 6.3.1 in Vietnam- Draft Final Report. 2018, March.

(19) Murto, Marika, L. Björnsson, and B. Mattiasson. 2004. "Impact of Food Industrial Waste on Anaerobic Co-Digestion of Sewage Sludge and Pig Manure." Journal of Environmental Management 70 (2): 101–7. https://doi.org/10.1016/j.jenvman.2003.11.001.

(20) MCDC (Mandalay City Development Committee), 2014. Water Quality Analysis Results Report. MUSIP (Mandalay Urban Services Improvement Project). Mandalay.

(21) Polprasert, C., 2007. Organic Waste Recycling - Technology and Management. 3rd Edition. IWA Publishing. Chapter 2 Characteristics of organic wastes. Pg-25.

(22) Won, S., Shim, S., You, B., Choi, Y., Ra, C., 2017. Nutrient production from dairy cattle manure and loading on arable land 30, 125–132.

(23) Giang, P. H., Harada, H., Fujii, S., Lien, N. P. H., Hai, H. T., Tanaka, S., & Kunacheva, C. (2012). Waste and wastewater management and its impacts in a sub-urban community: a nitrogen and phosphorus flow analysis in Hanoi, Vietnam. Japan Society of Civil Engineers, G(68(7)), III_741-III_749. https://doi.org/10.220AQ8/jscejer.68.III.

(24) MUSIP (Mandalay Urban Services Improvement Project). 2015. "TA-8472 MYA: Preparing Mandalay Urban Services Improvement Project Final Report, Volume 5, Wastewater FSR & Preliminary Design" 5 (April). Mandalay, Myanmar.

(25) DOP (Department of Population) 2015. The republic of the Union of Myanmar. "The 2014 Myanmar Population and Housing Census" report. vol 3-1 (May).

(26) Harada, H.; Dong, N. T.; Matsui, S. A Measure for Provisional-and-Urgent Sanitary Improvement in Developing Countries: Septic-Tank Performance Improvement. Water Sci. Technol. 2008, 58 (6), 1305–1311. https://doi.org/10.2166/wst.2008.715.

(27) Harada, H.; Schoebitz, L.; Strande, L. SFD Promotion Initiative – Danang. Vietnam. Report. 2015.

(28) Nakamura M, Yuzuyama Y (2005) Development of biomass database. Noukoukengihou 203:57–80 (in Japanese)

(29) Mosier, A., Kroeze, C., Nevison, C., Oenema, O., Seitzinger, S., 1998. Closing the global N 2 O budget : nitrous oxide emissions through the agricultural nitrogen cycle inventory methodology 225–248.

(30) Busser S, Nga PT, Morel A, Anh NV (2006) Characteristic and quantities of domestic wastewater in urban and peri-urban households in Hanoi. In: Proceedings of the Environmental Sci- ence and Technology for Sustainability of Asia, The 6th General Seminar of the Core University Program, Kumamoto.

(31) Parker, D. B. T. A. U.; Brown, M. S. T. A. U. Water Consumption for Livestock and Poultry Production. Conserv. Agric. 2003, No. February, 181–190. https://doi.org/10.1081/E-EWS.

(32) Hart, S. 2008. Meat Goat Nutrition. Pages 58-83 in Proc. 23rd Ann. Goat Field Day, Langston University, Langston, OK.

(33) Iannetta, P. P. M.; Young, M.; Bachinger, J.; Bergkvist, G.; Doltra, J.; Lopez-Bellido, R. J.; Monti, M.; Pappa, V. A.; Reckling, M.; Topp, C. F. E.; Walker, R. L.; Rees, R. M.; Watson, C. A.; James, E. K.; Squire, G. R.; Begg, G. S. A Comparative Nitrogen Balance and Productivity Analysis of Legume and Non-Legume Supported Cropping Systems: The Potential Role of Biological Nitrogen Fixation. Front. Plant Sci. 2016, 7 (November). https://doi.org/10.3389/fpls.2016.01700.

(34) Li, X.; Sørensen, P.; Li, F.; Petersen, S. O.; Olesen, J. E. Quantifying Biological Nitrogen Fixation of Different Catch Crops, and Residual Effects of Roots and Tops on Nitrogen Uptake in Barley Using in-Situ15N Labelling. Plant Soil 2015, 395 (1–2), 273–287. https://doi.org/10.1007/s11104-015-2548-8.

(35) Liu, J.; You, L.; Amini, M.; Obersteiner, M.; Herrero, M.; Zehnder, A. J. B.; Yang, H. A High-Resolution Assessment on Global Nitrogen Flows in Cropland. Proc. Natl. Acad. Sci. 2010, 107 (17), 8035–8040. https://doi.org/10.1073/pnas.0913658107.

(36) Shrestha, R. K.; Maskey, S. L. Associative Nitrogen Fixation in Lowland Rice. Nepal Agric. Res. J. 2010, 6 (0), 112–121. https://doi.org/10.3126/narj.v6i0.3373.

(37) Pharino, C., Sailamai, N., and Kamphaengthong, P.L. (2016). Material Flow Analysis of Nitrogen in Maeklong River Basin in Ratchaburi and Samut Songkhram Province, Thailand. Water Conservation Science and Engineering 1 (3)., 167–75. https://doi.org/10.1007/s41101-016-0011-1.

(38) IPCC (2000) Good practice guidance and uncertainty manage- ment in national greenhouse gas inventories. IPCC National Greenhouse Gas Inventories Program

(39) USDA (United States Department of Agriculture). USDA Food Composition Databases. Retrieved by April, 2018, from https://ndb.nal.usda.gov/ndb/search/list?home=true.

(40) Smil, V. Nitrogen in Crop Production : An Account of Global Flows Adds Recycled in Organic up by Harvested and Quantification of N Losses from Crop to 26-60. Global Biogeochem. Cycles 1999, 13 (2), 647–662. https://doi.org/10.1029/1999GB900015.

(41) Daioglou, V.; Stehfest, E.; Wicke, B.; Faaij, A.; van Vuuren, D. P. Projections of the Availability and Cost of Residues from Agriculture and Forestry. GCB Bioenergy 2016, 8 (2), 456–470. https://doi.org/10.1111/gcbb.12285.

(42) Schaffner, M.; Bader, H. P.; Scheidegger, R. Modeling Non-Point Source Pollution from Rice Farming in the Thachin River Basin. Environ. Dev. Sustain. 2011, 13 (2), 403–422. https://doi.org/10.1007/s10668-010-9268-2.

(43) FAO (2018) AQUASTAT database. http://www.fao.org/aquastat/en/countries-and-basins/country-profiles/country/MMR. Accessed April, 2018.

(44) JICA Expert Team, 2016. Water quality survey results report. Project for Capacity Development in Basic Water Environment Management and EIA System. MCDC. Mandalay

(45) Takamura Y, Tabuchi T, Harigae Y, Otsuki H, Suzuki S, Kubota H (1976) Studies on balance sheets and losses of nitrogen and phosphorus in the actual paddy field in the Shintone River basin. Jpn J Soils Sci Plant Nutr 48(9–10):431–436

(46) Takeuchi M (1997) Nitrate and phosphate outflow from arable land. Jpn J Soils Sci Plant Nutr 68(6):708–715

(47) Vries, W. De; Leip, A.; Reinds, G. J.; Kros, J.; Lesschen, J. P.; Bouwman, A. F. Comparison of Land Nitrogen Budgets for European Agriculture by Various Modeling Approaches. Environ. Pollut. 2011, 159 (11), 3254–3268. https://doi.org/10.1016/j.envpol.2011.03.038.

(48) ISD (Industrial Supervision Department), 2017. List of private industrial enterprises in the Mandalay industrial zones. (October) (in Burmese)

(49) James. I.Chang, and Tin-En. Hsu. 2008, "Effects of compositions on food waste composting". Journal of Bioresource Technology 99 (2008) 8068-8074

(50) Sokka, Laura, Riina Antikainen, and Pekka Kauppi. 2004. "Flows of Nitrogen and Phosphorus in Municipal Waste: A Substance Flow Analysis in Finland." Progress in Industrial Ecology 1 (1–3): 165–86. https://doi.org/10.1504/PIE.2004.004677.

(51) Zhang, Ruihong, Hamed M. El-Mashad, Karl Hartman, Fengyu Wang, Guangqing Liu, Chris Choate, and Paul Gamble. 2007. "Characterization of Food Waste as Feedstock for Anaerobic Digestion." Bioresource Technology 98 (4): 929–35. https://doi.org/10.1016/j.biortech.2006.02.039.

(52) MCDC. (2018). List of Market names in each township under Market and Slaughter Department, MCDC. received on 5th March,2018. (in Burmese)

(53) WHO, 1993. assessment of sources of air, water and land pollution report. Geneva.

(54) Mori, H., 2003. Technology Transfer Manual of Industrial Wastewater Treatment. Green Sustain. Chem. 3, 2–16. https://doi.org/10.4236/gsc.2013.32012

(55) JSWA (Japan Sewage Works Association) (1999). Survey on Sewage Development Comprehensive Plan for watershed areas: Guidelines and Explanation. (Ryuiki betsu gesuidou seibi sougou keikaku chousa: Shishin to kaisetsu) (in Japanese)

(56) MOI (Ministry of Industry), 2016. Industry Policy. February 2016. The government of the republic of the union of Myamar.

(57) MCDC (Mandalay City Development Committee), 2016, Urban Services Business Operation Plan for Solid Waste Management Report. MCDC, Mandalay.
